# Recent research advances on corrosion mechanism and protection, and novel coating materials of magnesium alloys: a review

**DOI:** 10.1039/d2ra07829e

**Published:** 2023-03-14

**Authors:** Liangyu Wei, Ziyuan Gao

**Affiliations:** a School of Material Science and Engineering, University of Science and Technology Beijing Beijing 100083 China; b Central Research Institute of Building and Construction (CRIBC) Beijing 100088 China gzyhhht0818@163.com +86 18969880147; c State Key Laboratory of Iron and Steel Industry Environmental Protection Beijing 100088 China

## Abstract

Magnesium alloys have achieved a good balance between biocompatibility and mechanical properties, and have great potential for clinical application, and their performance as implant materials has been continuously improved in recent years. However, a high degradation rate of Mg alloys in a physiological environment remains a major limitation before clinical application. In this review, according to the human body's intake of elements, the current mainstream implanted magnesium alloy system is classified and discussed, and the corrosion mechanism of magnesium alloy *in vivo* and *in vitro* is described, including general corrosion, localized corrosion, pitting corrosion, and degradation of body fluid environment impact *etc.* The introduction of methods to improve the mechanical properties and biocorrosion resistance of magnesium alloys is divided into two parts: the alloying part mainly discusses the strengthening mechanisms of alloying elements, including grain refinement strengthening, solid solution strengthening, dislocation strengthening and precipitation strengthening *etc.*; the surface modification part introduces the ideas and applications of novel materials with excellent properties such as graphene and biomimetic materials in the development of functional coatings. Finally, the existing problems are summarized, and the future development direction is prospected.

## Abstract 2

The performance of magnesium alloy implants has been continuously improved in recent years. This review discusses the types, corrosion mechanisms and corrosion control of medical magnesium alloys in recent years.

## Graphical abstract

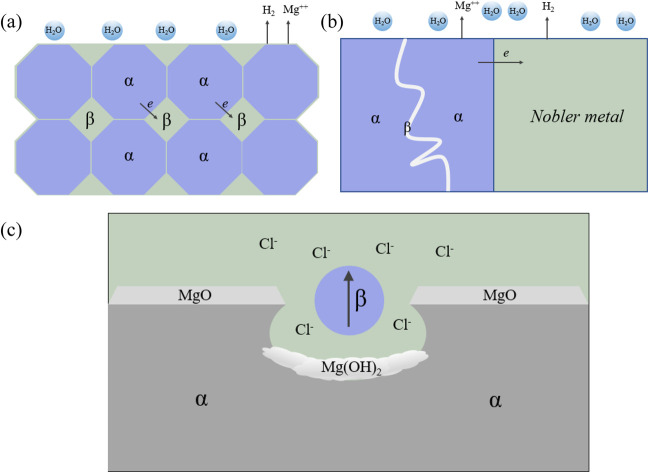

## 1. Introduction

The history of magnesium as a biomedical material can be traced back to 1878. Dr Huse used magnesium wire to suture a patient's blood vessels. However, since the rapid degradation rate of pure magnesium in the body could not be effectively controlled at that time, pure magnesium gradually faded out of the field of biomedical materials.^1^ Magnesium alloys have great application potential in the field of biomedicine due to their mechanical properties and degradability. By alloying with a variety of metal elements of different groups, a huge magnesium alloy system has been developed and widely used in cardiovascular stents, bone implant materials (bone nails, bone plates, *etc.*) and other fields.^2^ However, controllable degradation is the most basic and critical issue for medical magnesium alloys. The balance between local corrosion factors, mechanical strength, biodegradation rate and biocompatibility is still a research hotspot and difficult point in this field.^3^

As a biomedical implant material, magnesium alloy has both unique advantages and some disadvantages. Some properties of magnesium its alloys make them suitable for application in the biomedical field.

(1) Mg is an essential mineral for the human body. The recommended daily intake of magnesium for adults is 300–420 mg, and the concentration of magnesium in serum is 0.7–1.1 mmol L^−1^.^4^ Magnesium participates in a large number of human physiological activities, such as affecting the activity of more than 300 kinds of enzymes, maintaining a low resting calcium ion concentration in cells, cellular energy metabolism, protein synthesis, *etc.*^5^

(2) Mg has better mechanical properties: as shown in Table 1, compared with DL-PLA, mg alloys have higher mechanical strength; compared with inert metal materials such as 316L stainless steel, Co–Cr–(Ni)–Mo and Ti_6_Al_4_V, the elasticity modulus of Mg and its alloys (41–45 GPa) is closer to that of cortical bone (5–23 GPa), effectively reducing the stress shielding effect; Mg is a particularly light metal, with a density of 1.74 g cm^−3^, which is 64% of that of aluminum and 22% of that of steel, and this density is very close to that of human bone (1.75 g cm^−3^). The fracture toughness of Mg is greater than that of ceramic biomaterials such as hydroxyapatite, while the compressive yield strength of magnesium alloy (21–170 MPa) is closer to that of cortical bone (105–115 MPa) than other listed metal implants. Mg alloys have a large range of ultimate tensile strength and elongation, from 86 to 280 MPa and from 3% to 12%, respectively, which results in a rapid loss of strength during early degradation stages *in vivo*. The intrinsic strength of magnesium alloy as a stent material is not ideal. However, internal fixation does not require high strength or stiffness as it provides only temporary support.^6–15^

**Table 1:** Summary of physical and mechanical properties of common alloy implant materials compared with natural bone^6–15^

Tissue/material	Density (g cm^−3^)	Elastic modulus (GPa)	Tensile strength (MPa)	Yield strength (MPa)	Compressive strength (MPa)	Elongation	Ref.
Cortical bone	1.8–2.0	5–23	35–280	104.9–114.3	164–240	1.07–2.10	6
Cancellous bone	1.0–1.4	0.01–0.57	1.5–38	—	—	—	6
Mg–Zn–Ca–(Sr)	1.74–2.0	41–45	87–300	21–100	40–140	12	7
316L SS	7.9–8.0	190–205	540–1000	170–300	480–620	40	8
WE43	1.84	44.2	280	170	195	10	9
AZ91D	1.81	45	230	150	160	3	10
Ti_6_Al_4_V	4.43	110–120	860–965	760–1103	896–1120	12	11
Mg-cast	1.74	41	86.8	20.9	—	13	12
Co–Cr–(Ni)–Mo	8.3–8.9	230–240	860–1540	500–1500	450–1000	—	13
AM60B	1.78	45	220	—	130	6–8	14
DL-PLA	—	1.9–2.4	29–35	—	—	—	15

(3) Biodegradability: the degradation products of magnesium alloy in the human body environment are Mg(OH)_2_ and H_2_, which are harmless to the human body and will not introduce other harmful substances.^16^

Currently, large-scale application of Mg alloys in the clinical field still has the following obvious problems:

(1) Mg has a low standard potential of −2.37 V, which cannot generate an effective protective film to slow down the corrosion process. Corrosion continues to occur in the blood dynamic environment due to the inability to reach an electrochemical steady state.^17^

(2) Although the hydrogen as corrosion product of magnesium alloy is harmless to human body, cavitation which may delay wound healing and cause tissue necrosis, increasing the risk of blood flow blockage will be produced. The problem can be solved by subcutaneous puncture.^18^ Simultaneously, the rapid degradation of magnesium in the body is accompanied by an increase in the pH of local body fluids. The change of pH has potential harm to the growth of human bones and tissues, for example, it may cause the protein in human tissues to reach the isoelectric point and cause protein deposition or inflammation. In addition, hemolysis and local osteolysis may occur.^19^

The recent research focuses on improving the corrosion resistance of Mg and its alloys and expanding the biomedical field of Mg alloys. The following areas are more frequently reported: (1) preparation of new magnesium alloys by alloying; (2) surface treatment or deformation processing of existing magnesium alloys; (3) research and development of new forms of magnesium alloys (ultra-fine grain structure, glass metal, *etc.*). The research on Mg-based metal implant materials focuses on binary systems and ternary systems, and gradually develops towards multi-component alloys.^20^

**Fig. 1:**
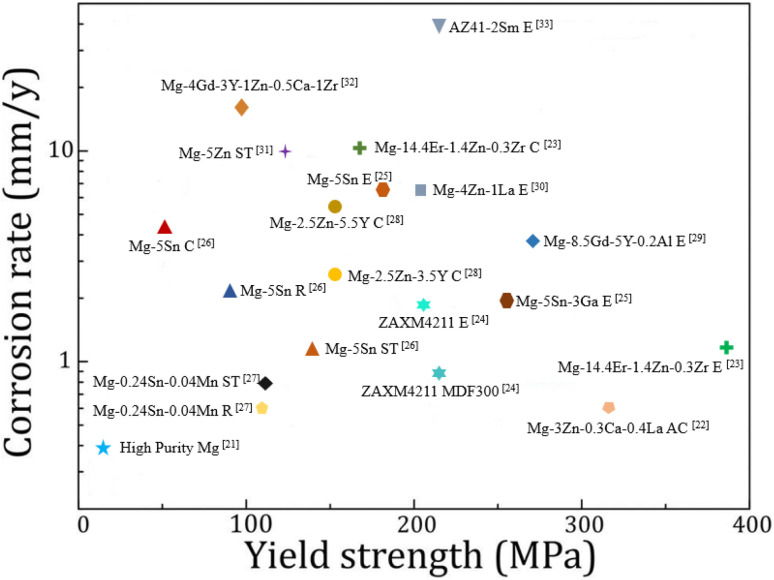
Corrosion rate *versus* yield strength of various magnesium alloys in 3.5 wt% NaCl solutions. The process name is C: casting, R: rolling, ST: solution treatment, MDF: multi-directional forging, AC: as-cast, E: extrusion.^21–33^

Heat treatment, plastic deformation and the addition of alloying elements are all common means to optimize the mechanical properties and corrosion resistance of magnesium alloys. A summary of the corrosion rate and tensile yield strength of recently reported magnesium alloys is shown in Fig. 1.^21–33^ It is not easy to achieve both corrosion resistance and mechanical properties by adjusting the type and quantity of alloying elements and changing the processing methods.

The active chemical properties of magnesium alloys cause an oxide film to form on the surface when exposed to air. The thickness of the oxide film formed in dry air is about 20–40 nm, showing an amorphous structure;^34^ the oxide film formed in humid air (about 65% RH, 30 °C) has a double-layer structure, including the outer oxide layer and inner oxide layer;^35^ the oxide film formed in aqueous solution has a three-layer structure, including an outer oxide layer, an intermediate layer and an inner oxide layer. The outer oxide layer is a sheet structure, composed of Mg(OH)_2_ and a small amount of MgO, with a thickness of about 1.8–2.2 μm. The structure of the middle layer and the inner oxide layer is similar to the double layer structure formed in humid air.^36^ The Pilling–Bedworth ratio (oxide/metal volume ratio) of MgO/Mg was 0.81. Therefore, the MgO layer structure is loose.^37^ When water molecules are adsorbed on the surface of the MgO film, the water molecules will dissociate into OH^−^ and H^+^, leading to the surface hydroxylation of MgO, which in turn forms a fragile loose oxide film Mg(OH)_2_. It is unstable in aqueous solution and is easy to dissolve the MgO/Mg(OH)_2_ film, exposing the metal to the solution and causing localized corrosion.^38,39^

Due to the existence of diverse and complex corrosion mechanisms, clarifying the corrosion mechanism of Mg and its alloys in the human body plays a vital role in solving the problem of excessive and uncontrollable corrosion rates. The scale of research on the *in vivo* corrosion behavior of Mg alloys is limited, and there is no unified conclusion on the factors affecting the corrosion of magnesium alloys, such as grain size, composition and distribution of second phase. Chemical experiments, immersion experiments or implantation experiments are carried out in an intuitive way, and part of their conclusions is contradictory, therefore, special analysis should be carried out in some special cases.^40^

Although alloying and deformation processing can significantly improve corrosion resistance, implants put forward higher requirements. Therefore, surface modification has become an idea that is expected to further break through the corrosion resistance limit of Mg alloys. The surface properties (morphology, microstructure and composition) of implants will regulate the adhesion of different cells, and thus the growth of different tissues.^41^ The basic principle of surface modification is to add a barrier layer between the Mg alloy and the corrosive medium through certain technical means, to reduce the corrosion tendency thermodynamically, and to hinder the diffusion of the corrosive medium kinetically. Surface coatings can be divided into inorganic and organic coatings. Common surface modification methods for preparing inorganic coatings include chemical conversion, anodization, micro-arc oxidation, chemical deposition, ion implantation and sol–gel. Organic coatings include degradable polymer coatings, and the common preparation methods include dip coating, spin coating and self-assembly.^42^ The formed inorganic coatings or degradable polymer coatings on the surface of Mg and its alloys can improve the corrosion resistance of magnesium alloys, which is conducive to maintaining the structure and performance of magnesium alloy implants in the early stage of implantation stability.^43^

Graphene materials have been widely used in the field of surface protection due to their excellent impermeability, oxidation resistance, high strength and lubricity. Graphene oxide (GO) has abundant oxygen-containing functional groups, which overcomes the problems of high chemical inertness and easy aggregation of graphene. In the past five years, graphene-based single-layer coatings, graphene/organic composite anti-corrosion coatings, graphene self-assembled coatings and multifunctional coatings have been reported. The emergence of graphene may break the performance and functional limitations of coating materials, however, the degree of crosslinking, bonding, the insertion of the cerium-based interlayer, the pre-alkali reduction of GO and the post-heat treatment reduction of the coating will all have an impact on the microstructure and corrosion and wear resistance of the biomimetic coating, which needs further discussion.^44^

Many properties of biomimetic materials broaden the design ideas of anti-corrosion coatings. Inspired by many natural species such as desert beetles, lotus leaves, and butterfly wings, the preparation of artificial superhydrophobic coatings has been rapidly developed.^45–47^ The two key factors for the preparation of superhydrophobic surfaces are chemical composition energy with low surface energy and suitable surface micro–nano hierarchical structure.^48^ The application of the superhydrophobic coating can reduce the contact area between the corrosive liquid and the surface to inhibit the corrosion development of the magnesium alloy in the solution environment.^49^ The excellent adhesion of polydopamine (PDA) coatings inspired by the strong adhesion of marine mussels provides multiple possibilities for subsequent surface modification, such as hydrophobic monolayers and protein adsorption.^50^ Similar biomimetic materials are showing potential as functional coatings for implanted magnesium alloys.

Based on the above research hotspots and trends, this review will start with the introduction of existing medical Mg alloy systems and their applications that have been widely reported in recent years, and then discuss the corrosion mechanism, influencing factors and evaluation methods of magnesium alloys *in vivo* and *in vitro*. Next, methods for improving the corrosion resistance of Mg alloys are introduced: alloying and surface treatment, and some new coating materials with impressive characteristics will be inventoried. Finally, the problems to be overcome are summarized, and the outlook is put forward.

## 2. Magnesium alloy system classified from the perspective of human body elements

### 2.1 Mg alloy developed based on essential elements of life

**Fig. 2:**
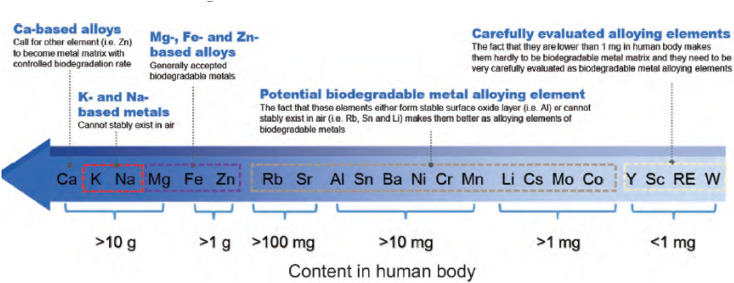
Contents of metal elements in human body and suggestions on alloying of each element.^51^

From Fig. 2,^51^ the intake of metal elements in the human body needs to be strictly assessed. The total content of important physiological elements Ca, K, Na, Mg, Fe, Zn in the human body exceeds 1 g, which can be prioritized as alloying elements. Ca, K and Na are very active and cannot exist stably in the air, thus they are not suitable as matrix components, but can be added as alloying elements. During the design of the implant, take a bone nail as an example, assuming its weight is about 1 g, if the elements Li, Cs, Mo and Co are used as alloying elements (the total amount in the human body is between 1 and 10 mg), the bone when the material used for the nail is added with a mass fraction of 0.1%, the amount of such elements introduced into the human body through the implant is 1 mg, which is close to the total intake of the human body, so vigilance is required. For elements such as Sc and Re, the human intake is less than 1 mg, and it is easy to exceed the human intake after degradation, strict biosafety evaluation (pharmacokinetics, toxicokinetics, *etc.*) is needed before clinic trail.

Based on this principle, a series of alloys have been issued, including a series of typical binary alloys such as Mg–Ca, Mg–Zn, Mg–Mn, Mg–Li, Mg–Sr, Mg–Si, *etc.*

Mg–Zn alloys have good plasticity and biocompatibility. Frequently reported models are: Mg–6Zn, Mg–Zn–Zr, Mg–Zn–Ca, Mg–Zn–Fe, Mg–Zn–Ca–Fe, Mg–Zn–Mn–Ca, Mg–Zn–Mn–Ca–Fe, *etc.* Ternary and quaternary alloys containing rare earth elements include Mg–Zn–Y (such as WZ21 (2 wt% Y, 1 wt% Zn) and ZW21 (2 wt% Zn, 1 wt% Y)), Mg–Zn–Gd, Mg–Zn-*x*-RE (such as ZEK100 (1 wt% Zn, 0–0.5 wt% Zr, 0–0.5 wt% RE)), *etc.*^52^ The addition of rare earth elements forms a uniform microstructure with uniform grain size and dispersed second phase composition, which effectively improves the mechanical properties of the alloy and reduces the degradation rate. Special phases in Mg–RE alloys, such as the long-period stacked order (LPSO) phase (Mg–Zn–Y), show high strength, usable ductility and good corrosion resistance.^53^

The amount of Sr added in Mg–Sr binary alloys is generally 0.3 wt% to 4 wt%. The Mg–Sr alloy is mainly composed of α-Mg and Mg_17_Sr_2_ phases, and galvanic corrosion exists between the two phases, which accelerates the degradation. The addition of 1.5–2 wt% Sr helps to improve the corrosion resistance, while further addition will have the opposite effect due to the increase of the second phase and the formation of galvanic cells.^54^ After *in vivo* and cytotoxicity tests on Mg–0.5Sr,^55^ Mg–1.5Sr^56^ and Mg–2Sr,^57^ they showed that the cell viability of the samples after 7 days was higher than that of the control group WE43; also, they had good osteogenesis capacity, slight host reaction and safe residual Sr element for human body after 4–8 weeks of implantation. In short-term *in vivo* experiments, Mg–Sr alloys exhibit good biocompatibility and bioactivity, making them promising for orthopaedic applications. Long-term *in vivo* experiments are needed to verify the reliability of these properties.

**Fig. 3:**
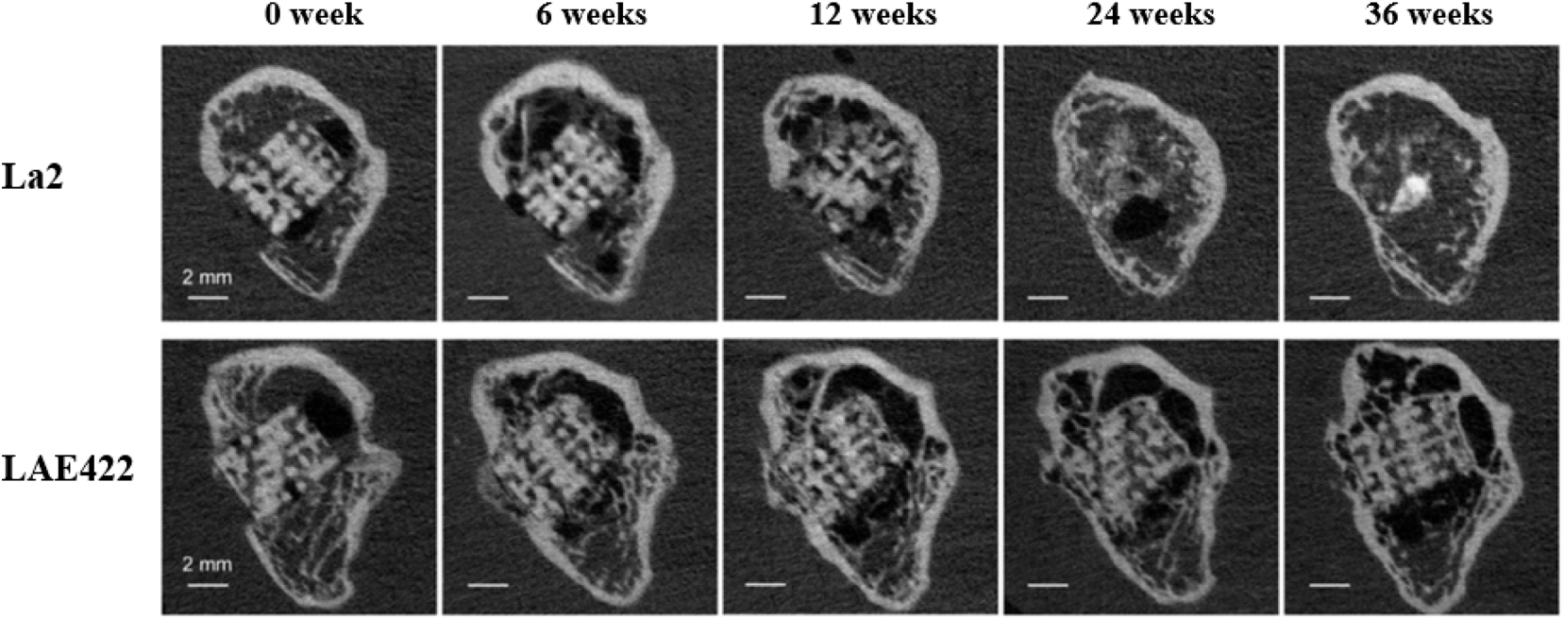
μCT images of longitudinal sections of La2 and LAE422 degradation evolution at 36 weeks.^60^

The Mg–Li binary alloy has good plasticity, but the a lower strength compared to pure Mg. Al, Ca and Y are effective alloying elements for this type of alloy, and ternary and quaternary alloy systems have been developed. Typical Mg–Li ternary alloy systems include Mg–Li–Al: LA33, LA63, LA93; Mg–Li–Ca: Mg–1Li–1Ca, Mg–4Li–1Ca, Mg–9Li–1Ca and LAE442 (4 wt% Li, 4 wt% Al, 2 wt% RE). LAE442 has been tested in various *in vivo* studies such as dowel, full body cylinder or plate screw systems, and a small number of stents. Processing the LAE422 alloy by severe plastic deformation represents an option to improve its mechanical properties and corrosion resistance.^58^ In the microscopic observation of LAE442 treated with equal channel angular pressure (ECAP), FCC nano-aluminum particles were formed in the magnesium matrix, and the Al at dislocations was strongly segregated. The ECAP-treated alloys were tested for *in vitro* degradation in physiological saline, and their degradation rates decreased significantly. *In vitro* cytotoxicity tests showed that the 7 day viability of ECV304 and VSMC cells could reach 80% for the ECAP-treated material.^59^ In *in vivo* tests with Mg–La2 stents, LAE442 showed a homogeneous and slower degradation and achieved better osseointegration values in the first few weeks. Due to the excessively fast degradation rate, the BMD, bone volume, trabecular number and thickness displayed by La2 were smaller than those of LAE422, and the value of trabecular spacing was also larger. Although bone growth conditions were improved after almost complete degradation of La2 and cessation of gas production, the overall growth quality was still suboptimal due to the fast degradation (Fig. 3).^60^

### 2.2 Industrial magnesium alloy

Some implant Mg alloys can be transformed from industrial magnesium alloys. The research of these alloy system mainly focuses on Mg–RE system and Mg–Al based magnesium alloys. Binary Mg–RE alloys containing rare earth elements lanthanum (La), cerium (Ce), neodymium (Nd), gadolinium (Gd), dysprosium (Dy), yttrium (Y) and scandium (Sc) have been widely reported. The addition of these elements has played a role in grain refinement, improving the corrosion resistance and mechanical properties. The binary RE phase diagrams is similar for the Mg-rich regions, they all have simple eutectic reactions to form Mg_24_Y_5_ or Mg_12_RE.^61^

Although a large number of research reports, currently, the only Mg–RE implants with valid clinical trial data are the MAGNEZIX series of orthopaedic prosthetic devices (screws, pins and arthrodesis) by Syntellix AG and the Magmaris series stent (DREAMS-2G) by Biotronik.

#### 2.2.1 Mg–RE alloy as a cardiovascular stent

Among the Mg–RE alloys, the Mg–Y–RE–Zr quaternary alloy WE43 is more representative. Screws, pins and brackets made of WE43 (W for Y element, E for RE element, 4 wt% Y, 3 wt% RE) are currently receiving extensive clinical and testing. Gualter *et al.* evaluated the corrosion performance of WE43 in 0.6%NaCl solution and found that the precipitation second phase was Mg_41_Nd_5_ and Mg_24_Y_5_, and the corrosion resistance was greatly improved compared with pure Mg. In the later stage of corrosion, the protective corrosion product layer composed of Mg(OH)_2_, Y_2_O_3_ and Nd_2_O_3_ covers the surface of the alloy, and at the same time hinders the charge transfer between precipitate and matrix, making corrosion uniform and slow.^62^ However, the biocompatibility issues brought about by the rare earth elements introduced by the alloying of WE43 need further long-term evaluation.

Biotronik developed a degradable Mg alloy stent based on WE43 and carried out technical updates, its stents have undergone the evolution from AMS to DREAMS. After three iterations, the radial support force was further enhanced while the degradation rate was reduced, and the intimal hyperplasia was reduced by improving the drug-loaded coating.

In 2005, Peeters *et al.* treated with Biotronik's AMS stent and found symptoms of significant limb ischemia. Three-month follow-up showed a cure rate of 89.5% (17/19); the stent was completely degraded after six weeks.^63^ The degradation rate is fast and cannot meet the needs of rehabilitation. In view of this, AMS's improved DREAMS 1-G stent adopts optimized alloy composition and structural design to further enhance radial support force and reduce degradation rate, and prepare paclitaxel-loaded PLGA coating on the stent surface to reduce intimal hyperplasia. The clinical trial of BIOSOLVE-I showed that DREAMS 1-G had good safety after implantation. The angle of the blood vessel at the implanted position recovered at 6 months, indicating that the stent had lost its supporting function. The IVUS results also showed that the stent had significantly degraded at 6 months. There was no difference in in-stent LLL (0.65 ± 0.50 mm) at 6 months and results at 12 months (0.52 ± 0.39 mm). Although both were significantly lower than the 4 month results for the AMS stent (1.08 ± 0.49 mm), they were still significantly higher than the conventional DES stent.^64^ In order to further reduce the in-stent LLL of DREAMS 1-G, DREAMS 2-G takes PLLA as the drug-loading layer, it contains rapamycin which is generally believed to have better anti-intimal hyperplasia effect. X-ray markers made of tantalum is placed at both ends of the stent for more accurate positioning. The clinical trial results showed that the target lesion failure rate (TLF) of the DREAMS 2-G was only 3.3% after 6 months of implantation, and no definite or probable stent thrombosis occurred. The in-stent LLL at 6 months was (0.44 ± 0.36) mm, which was lower than the in-stent LLL at 6 months of DREAMS-1G (0.65 ± 0.50 mm); the area of intimal hyperplasia (0.08 ± 0.09 mm^2^) was also lower than that of DREAMS 1-G (0.30 mm^2^).^65^ Based on the excellent clinical performance of DREAMS 2-G, the product has obtained CE certification in June 2016, which is also the world's first approved biodegradable Mg alloy vascular stent product (Table 2).

**Table 2:** Parameters of AMS and DREAMS stent^66^

Product name	AMS	DREAMS 1-G	DREAMS 2-G
Structure	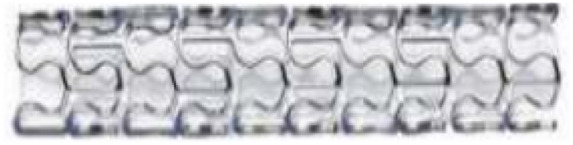	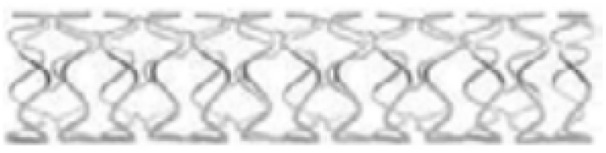	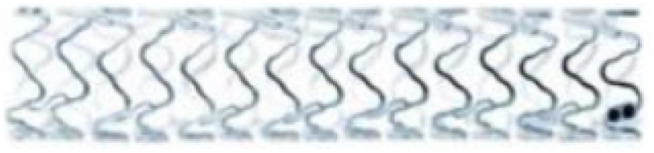
Matrix material	WE43	Refined magnesium alloy	Refined magnesium alloy
Dimension (μm)	80 × 165	130 × 120	150 × 150
Coating	—	PLGA	PLLA
Drug	—	Paclitaxel	Sirolimus
Design	4-Crown 4-link	6-Crown 3-link	6-Crown 2-link
X-ray markers	—	—	Tantalum marker on both ends
Implantation time	1 week	4 weeks	12 weeks

The main problems faced in the research of magnesium alloy vascular stents are as follows:

① The difference between *in vitro* and *in vivo* degradation rates. The degradation rate is one of the most important indicators of biomedical magnesium alloys, which not only determines the decay rate of mechanical properties, but also determines the local pH value and ion concentration of alloying elements at the implantation site, thereby affecting the biocompatibility. However, the degradation rates obtained from *in vitro* and *in vivo* experiments are usually inconsistent because *in vitro* experiments are difficult to simulate the real human environment. Usually, the simulated body fluid (SBF) used in experiments only contains inorganic salt ion concentrations (Na^+^, K^+^, Mg^2+^, Ca^2+^, Cl^−^, HCO_3_^−^, HPO_4_^2−^, SO_4_^2−^), while human blood also includes amino acids, proteins, lipids, cells and other components. Dulbecco's modified eagle's medium (DMEM) with inorganic salt ions and amino acid concentrations is more like human plasma and is recommended as a corrosion medium for *in vitro* experiments.^67^

The effect of protein on the degradation of magnesium alloys is not well established, as it may inhibit or promote the degradation, depending on the type of protein and the properties of the alloy surface. Johnson *et al.* studied the effect of fetal bovine serum (FBS) on the degradation of pure Mg and MgY and found that FBS had no obvious effect on the degradation of pure Mg, (with or without oxide film) but accelerated the degradation rate of MgY (even with the protection of oxide film), which may be due to sequestration of metal ions and oxides or promotion of local galvanic cells.^68^ Liu *et al.* found that addition of albumin to normal saline could reduce the degradation rate of Mg–1.5Ca. Electrochemical impedance spectroscopy (EIS) tests showed that albumin could improve charge transfer resistance and surface layer resistance at open circuit potential, while at the same time the effect of albumin in reducing the degradation rate increases with increasing concentration.^69^ Other factors include the body's pH buffer system, fluid flow, substance transport, metabolism, cellular and tissue effects. Among them, the effect of cells on the degradation behavior of biomedical magnesium alloys is rarely reported.

Due to the biodegradation characteristics of magnesium alloys, the ISO10993 *in vitro* evaluation standard is not fully applicable to degradable magnesium alloys, and related experimental method improvement and standard formulation (including alloy preparation, material screening, and full-device performance testing, *etc.*) are still in progress.^70–72^

② The mechanism of further transformation and degradation products is not clear. The degradation process of polymer drug-eluting stents has been clearly studied. Taking the BVS 1.1 stent as an example, the degradation process of the PLLA stent includes autocatalytic ester bond hydrolysis, molecular weight reduction, and the diffusion of the hydrophilic water product lactic acid monomer into surrounding tissues and blood, and finally metabolized to CO_2_ and water through the pyruvate and tricarboxylic acid cycles. After 36 months, the stent was completely degraded and the stent site was occupied by collagen, proteoglycans and a small amount of smooth muscle cells.^73^

Compared with polymer vascular stents, the entire degradation process of Mg alloy vascular stents and the transformation and metabolism mechanism of degradation products have not been systematically studied. The results of the PROGRESS-AMS clinical trial showed that 4 months after the AMS stent was implanted, the echo reflection signal of the stent wall had been significantly reduced and there was no shadow caused by the metal when it was just implanted, but the wall could still be distinguished from the surrounding tissue. After 16 months of implantation, the reflected signal was no longer detectable. From this, it was indirectly inferred that the stent was completely degraded at 4 months, and there was no residue at the implantation site after 16 months.^74^

The degradation products of DREAMS-1 are mainly composed of Mg and O elements, which may be Mg(OH)_2_ or MgCO_3_. The DREAMS-1 degraded uniformly *in vitro*, and *in vivo* results showed that the degradation occurred preferentially on the side, which may be related to hemodynamics; after 90 days, the stent was significantly degraded, and the degradation products of the outer layer were converted into calcium phosphate; after 180 days, the magnesium alloy was completely degraded, the degradation products were completely converted to amorphous calcium phosphate.^75^

**Fig. 4:**
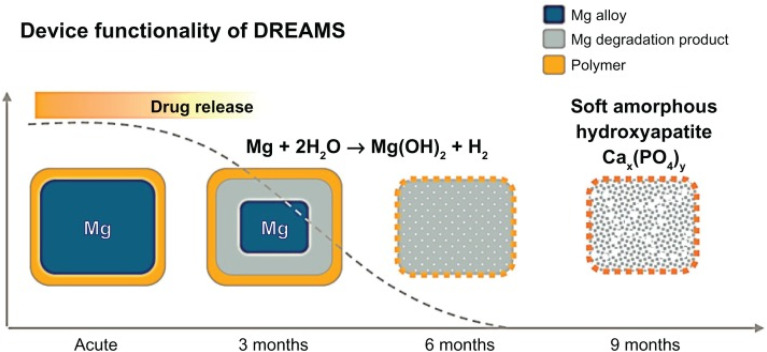
Degradation model of DREAMS-2 stent.^76^

The BIOSOLVE II clinical trial using the DREAMS-2 stent was reported as one of the themes at the 2015 TCT (Transcatheter Cardiovascular Therapeutics of the Cardiovascular Research Foundation) conference. Degradation and absorption model of DREAMS-2 stent magnesium (Fig. 4): in the early stage after implantation, Mg and H_2_O react to form Mg(OH)_2_ (the blue part of the stent periphery); 3–12 months later, there are Mg(OH)_2_ around the degradation product with the deposition of phosphates, Mg(OH)_2_ is gradually converted into more insoluble Mg(H_*x*_PO_4_)_*x*_ (the cyan part of the periphery of the stent), and due to the deposition of Ca, Mg(H_*x*_PO_4_)_*x*_ is gradually converted into Ca(H_*x*_PO_4_)_*x*_; after 12 months, the degradation products are completely converted to Ca(H_*x*_PO_4_)_*x*_, and Mg ions diffuse into the blood and finally excrete through the urine.^76^

#### 2.2.2 Mg–RE alloy as bone fixation materials

**Fig. 5:**
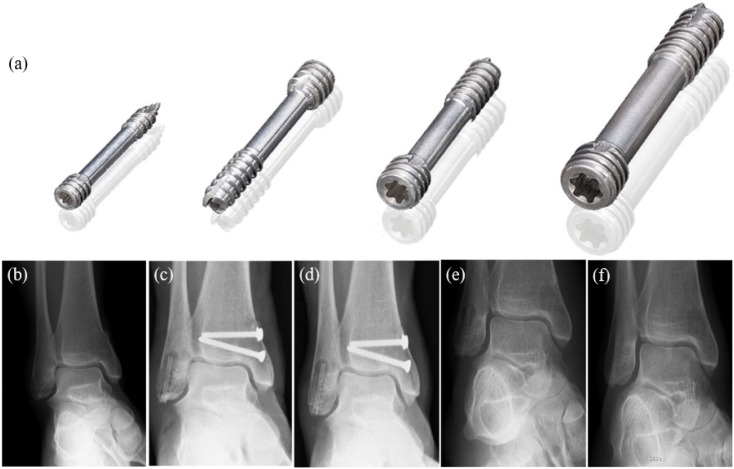
(a) MAGNEZIX® compression screws: from left to right: MAGNEZIX® CS 2.0, MAGNEZIX® CS 2.7, MAGNEZIX® CS 3.2, MAGNEZIX® CS 4.8. (b–f) X-ray of ankle fracture: (b) ankle fracture type AO-ASIF 44-A2.3; before surgery (c) six weeks after surgery; (d) 3 months after surgery; (e) 8 months after surgery, metal removal; (f) 17 months after surgery.^81^

Because the elastic modulus of magnesium alloy is close to that of human bone, it can effectively alleviate the stress shielding effect. Magnesium alloys can provide stable mechanical properties in the early stage of fracture healing, gradually reduce its stress shielding effect, and prevent the occurrence of local osteoporosis and refractures.^77^ The bone fixation materials involved in the experiment mainly include alloy rods, metal laths and screws, *etc.* Although many Mg alloys have been applied to orthopedic research, only the MAGNEZIX cannulated compression screw from Germany's SYNTELLIX company has obtained EU certification, and the K-MET cortical bone screw and headless screw from South Korea's U&i company have obtained marketing approval from the Korean Food and Drug Administration.^78^ MAGNEZIX® is an absorbing compression screw modified from Mg–Y–RE–Zr alloy and is the first implant of its kind to receive CE certification. These products have a yield strength (YS) greater than 260 MPa, an ultimate tensile strength (UTS) greater than 290 MPa, an elongation of 8%, and an elastic modulus of approximately 45 GPa. The screw has been developed in different models with diameters of 2.0 mm, 2.7 mm, 3.2 mm and 4.8 mm, and thousands of them are sold worldwide.^79^ Waizy *et al.* conducted an *in vivo* study in New Zealand white rabbits using Mg–Y–RE–Zr, which has a similar composition to WE43. μCT-stained tissue showed essentially complete degradation of the metal portion of the implant 52 weeks after implantation.^80^ The Nuremberg General Hospital reported a case of postoperative medial malleolus fixation with MAGNEZIX® CS 3.2 in a 43 year-old patient with a bi-ankle fracture.^81^ From Fig. 5, at 6 weeks post-operatively (Fig. 5(c)), a distinct radiolucent area appeared in the proximal end of the MAGNEZIX® screw. At 3 months postoperatively (Fig. 5(d)), the radiolucent area was unchanged. The degradable properties and good strength of Mg–Y–RE–Zr alloy provide reliable support for metal removal, allowing it to be removed smoothly without residue. After removal at the eighth month (Fig. 5(d)), the area of X-rays passing through the damaged area was greatly reduced, indicating that the bone rehabilitation was progressing smoothly. Fig. 5(f) shows the almost complete disappearance of X-ray transparency at 17 month postoperative follow-up. The patient had no pain, swelling, or other functional deficits. Although the light transmittance around the MAGNEZIX® screw deviates from previously reported osteoconductivity, results at both 8 and 17 months post-surgery demonstrated that the screw did not cause allergic reactions or other disturbances to bone healing.

Also, there are studies on the degradation behavior of WE54 magnesium alloy (die-cast, 1.58 wt% Nd, 4.85 wt% Y, 0.28 wt% Zr, 0.08 wt% Ce, 0.13 wt% Gd, 0.16 wt% Er, 0.13 wt% Yb). The degradation rate of the solutionised (200 μA cm^−2^) and peak-aged (160 μA cm^−2^) WE54 alloy is lower than that of pure magnesium (250 μA cm^−2^), but significantly higher than that of the AZ91 alloy (50 μA cm^−2^). The passivation film resistance formation of WE54 is not ideal.^82^ EW62 (Mg–6%Nd–2%Y–0.5%Zr) magnesium alloy has improved corrosion resistance and mechanical properties compared to its conventional counterpart (CC), and rapid solidification properties also reduce its cytotoxicity.^83^ Increased Nd solubility in passivated substrates and outer layers helps reduce hydrogen formation and hydrogen embrittlement.^84^ EW10X04 (1.16 wt% Nd, 0.48 wt% Y, 0.48 wt% Zr, 0.43 wt% Ca) developed by adding a small amount of Ca to EW10 (1.15 wt% Nd, 0.43 wt% Y, 0.46 wt% Zr), the corrosion test in 0.9% NaCl solution found that the corrosion rate of EW10X04 (about 0.5 mm year^−1^) was significantly slower than that of EW10 (more than 1 mm year^−1^), however, the mechanical properties of EW10X04 decreased.^85^

#### 2.2.3 Mg–Al alloy and unclassified modified Mg–RE alloy

Mg–Al alloys commonly used in industry are represented by as-cast AZ91 and extruded AZ80, AZ63, AZ31, AM60, AE21, *etc.* According to the Mg–Al alloy phase diagram, at 710 K, the solid solubility of Al in Mg reaches a peak of 12.7 wt%; when it drops to room temperature, the solubility of Al in Mg decreases to about 2 wt%, and the eutectic reaction is: L → α(Mg) + β(Mg_17_Al_12_). However, under actual solidification conditions, such as AZ91 magnesium alloy, a dissociated eutectic structure or a non-equilibrium eutectic structure will be formed.^86^ For AZ31, Al is completely dissolved in α-Mg without the formation of β-phase.^87^ Since excessive Al may cause neurotoxicity, which is closely related to the occurrence of Alzheimer's disease, Mg–Al medical magnesium alloys are gradually being replaced by other series of alloys.

##### 2.2.3.1 JDBM

Yuan *et al.* developed JDBM-1 for orthopedics with “high strength and moderate toughness” and JDBM-2 for cardiovascular use with “high plasticity and medium strength”, both of which are Mg–Nd–Zn–Zr quaternary alloy (Nd 3.0, Zn 0.2, Zr 0.5). The alloy adds a small amount of light RE element Nd with mild cytotoxicity. The addition of Nd can ensure that alloy has good aging precipitation strengthening and solid solution strengthening performance, also, it brings more uniform corrosion by greatly increasing the electrode potential of the alloy matrix and reducing the galvanic corrosion potential difference between the matrix and the second phase, the biocorrosion resistance and mechanical properties of JDBM in SBF is significantly better than that of AZ31 and WE43.^88^

In the evaluation of *in vitro* cytocompatibility, JDBM showed good compatibility with endothelial cells and smooth muscle cells, and had no adverse effect on the activity, apoptosis and cytoskeleton of HUVEC cells; adhesion morphology and apoptosis were not adversely affected. In *in vitro* test, the 72 h corrosion rate of JDBM stent was calculated to be (0.23 ± 0.02) mm y^−1^ by weight loss method. Proteins can reduce this corrosion rate by enhancing the protection of the degradation product layer, whereas HUVEC cells and macrophages can accelerate the degradation of JDBM, where the promotion by macrophages is uniform and does not accelerate localized corrosion.^89^ In the *in vivo* experiment (New Zealand white rabbit), the JDBM implantation process had good compliance, and the stent did not fall off from the balloon. JDBM adhered well after expansion and had good biosafety after implantation. Complete endothelialization of JDBM took 4 weeks and the progress was similar to that of 316L SS. It took about 3 months for Mg in JDBM to degrade completely. Mg and Zn showed no continuous enrichment in the main organs of rabbits. Part of Nd existed in the blood vessel wall in the form of Nd(OH)_3_ and Nd phosphate particles, the other part was metastasized to the spleen, liver and lung, and there was no persistent enrichment in the organs, which is a relatively safe signal. Several months after implantation, the content of Zr in the spleen and liver was higher than that in the control group without implantation. The problem JDBM needed to face is that stress corrosion occurs in only one month after implantation, resulting in a significant decrease in the support of the blood vessel. Related stent structure needs to be further optimized.^90^ Surface treatment technology may effectively improve this defect. It is currently reported that the Ca–P coating JDBM screw used for the treatment of fractures prolonged the complete degradation time to 3–6 months, and no infection, internal fixation failure, malunion or other complications occurred in any patient.^91^

### 2.3 Other medical magnesium alloys

Y, Sc and W are also trace elements in the human body, and their intake should be strictly evaluated. The application of their multi-component alloys in the field of implants has also been reported.

#### 2.3.1 Mg–Y

Compared with pure magnesium, the binary alloy Mg–Y has refined grains and improved corrosion resistance.^92^ Ternary alloy Mg–Y–Zn significantly improves the uniformity of degradation and biocompatibility.^93,94^ Quaternary alloy Mg–Y–Ca–Zr^95^, Mg–Y–RE–Zr^96^ significantly improves mechanical properties while maintaining *in vitro* cell compatibility.

#### 2.3.2 Mg–Sc

Ogawa *et al.* found that there is a bcc structure (β-type) in Mg–Sc alloys. The β-type Mg–Sc alloy caused reversible martensitic transformation and exhibit shape memory properties. Shape memory magnesium alloys are very suitable for stents.^97^ A study on Mg–3Sc–3Y found that the addition of Sc and Y could moderate the corrosion rate in the form of passive films. The oxide layer was mainly composed of Sc_2_O_3_ and Y_2_O_3_, and the content of Sc_2_O_3_ was higher than that of Y_2_O_3_. Compared to the polished surface, the oxide layer reduced the degradation rate by a factor of nearly 100 for up to 3 weeks. The compressive strength of this alloy also reached the level of AZ91 and WE43.^98^ Ternary Mg–Sc–Sr alloys showed significantly higher angiogenic and osteogenic differentiation compared to pure magnesium and controls, demonstrating potential for bone implants.^99^

#### 2.3.3 Mg–W

To date, there are few reports on multi-component medical alloys with Mg–W as the main alloying element. In view of the potential harm of W to the human body, it should be carefully evaluated.

The above discussion is all about the Mg alloy produced by alloying. Also, researchers use surface modification methods to build a corrosion barrier layer on the surface of Mg alloys for degradation regulation and control. Common methods mainly include the preparation of chemical conversion coatings, electrochemical coatings, Ca–P-based coatings, degradable polymer coatings, bioinert ceramic coatings and composite coatings.^100^ From this, a more diverse and huge medical magnesium alloy system is derived. Looking forward to more comprehensive and systematic reports in related fields in the future.

## 3. Corrosion mechanism of Mg alloys

### 3.1 Corrosion types of Mg alloys

Mg is an active metal element. In acid and neutral solutions, especially in solutions containing Cl^−^, Mg is prone to electrochemical corrosion after contacting with H_2_O, and Mg(OH)_2_ is formed on the surface and H_2_ is released at the same time. Human body fluid is a complex environment of a variety of anions, cells, proteins, glucose and pH, and the degradation is more complicated than that in the natural environment. According to the mainstream view, the explanation is as follows: (1) the oxide film and corrosion layer on the alloy surface are loose in structure and chemically unstable; (2) galvanic corrosion caused by impurities and secondary phases in the alloy.^101^

The corrosion types that are prone to occur in the aqueous solution are mainly general corrosion, pitting corrosion and localized corrosion. The process of general corrosion is uniform and occurs over the entire metal surface. This type of corrosion is less detrimental to the implant material and is common in the self-dissolution of pure metals and homogeneous alloys. During localized corrosion, the corrosion rate of local area of the alloy is much greater than that of the rest of the alloy, and the corrosion is more destructive than general corrosion. Common localized corrosion include intergranular corrosion, filiform corrosion, stress corrosion cracking and corrosion fatigue *etc.* (Table 3).^102^

**Table 3:** Common corrosion types of magnesium alloys in simulated physiological fluids^103–128^

Corrosion types	Corrosion morphology	Predisposing factors	Typical cases	Reference
General corrosion	Uniform corrosion characteristics	Aqueous electrolyte (chloride, perchlorate, sulfate, nitrate anions)	Almost all magnesium alloys, such as AZ91, AZ31, AE44, *etc.*	103–105
Pitting corrosion	Pits	Electrode potential difference, grain size, pH	Common in most magnesium alloys, such as AZ31, WE43, ZK60, ZE41, GW93, AM60, Mg–Zn–Y–Nd, Mg–Nb, Mg–La, Mg–Ca, Mg–Sr, Mg–Nb, Mg–Al–Ti, *etc.*	106–111
Localized corrosion: galvanic corrosion	Blowholes, cracks	Potential difference, pH, galvanic pair spacing	AZ91D, Mg–Dy–Gd–Zr, Mg–Gd–Ca, Mg–Y–Zn, Mg–Zn, Mg–Ca, *etc.*	112–117
Localized corrosion: intergranular	Pearly pitting, grain off	Microgalvanic corrosion induced	AZ80, ZK60, *etc.*	118 and 119
Localized corrosion: filiform corrosion	Corrosion wire on alloy surface	Originating from pitting corrosion, induced by corroded microbatteries	Mg–Li, Mg–Zn, WE43, *etc.*	120–122
Localized corrosion: corrosion fatigue	Fatigue crack	Microstructure, loading frequency, temperature, humidity, and pH	AZ91D, WE43, AZ80, ZX10, Mg–Zn–Zr–Y, *etc.*	123–126
Localized corrosion: stress corrosion cracking	Crack	Electrochemistry, tensile mechanics, alloying elements, pH	Al-containing magnesium alloy (AZ31, AZ61 and AZ91), ZK21, ZX10, *etc.*	126–128

#### 3.1.1 Galvanic corrosion

In NaCl solution, the corrosion potential of Mg is more than 600 mV negative than that of the second active engineering metal Zn, and Mg alloy implants are always quite active anodes when they are in contact with other implanted metals such as stainless steel and titanium alloys. Galvanic corrosion can occur between the matrix phase and the second phase even within the same material.^129^

Magnesium alloys are not uniform in composition, microstructure and crystallographic orientation. These differences lead to various electrochemical activities, resulting in microscopic-scale galvanic couples. The potential difference between the Mg alloy and the coupled metal is positively related to degradation rate when it is used as an anode. Therefore, when choosing a galvanic pair, making the potential difference between the coupling metal and Mg alloy less than 0.25 V. Changes in pH may alter the electrolytic reaction as well as the polarity of the galvanic metals. For example, in a neutral or weakly acidic low-concentration NaCl solution, Al–Mg galvanic couple is initially using Mg as anode, but with dissolution of Mg, the solution becomes alkaline, and Al is converted into anode. Therefore, in alkaline medium (pH > 8.5), the corrosion process of alloy is greatly inhibited.^130^

Anode/cathode area ratio, anode/anode insulation distance, depth of the dissolved film overlying the galvanic pair and distance between the galvanic pairs all affect the behavior of galvanic degradation. The galvanic current action distance of Mg alloys is limited, and corrosion generally occurs near the edge of test piece. Song *et al.* used a specially designed “sandwich” galvanic corrosion probe to measure galvanic currents at different distances between two pieces of metal, high-strength steel and AZ91D under standard salt spray conditions. From them, under theoretical relationship, the galvanic current decreases with the increase of distance between cathode and anode, and the relationship is exponential, and the larger the distance, the smaller the reduction. The experimental values are basically in line with the theoretical relationship.^117^

**Fig. 6:**
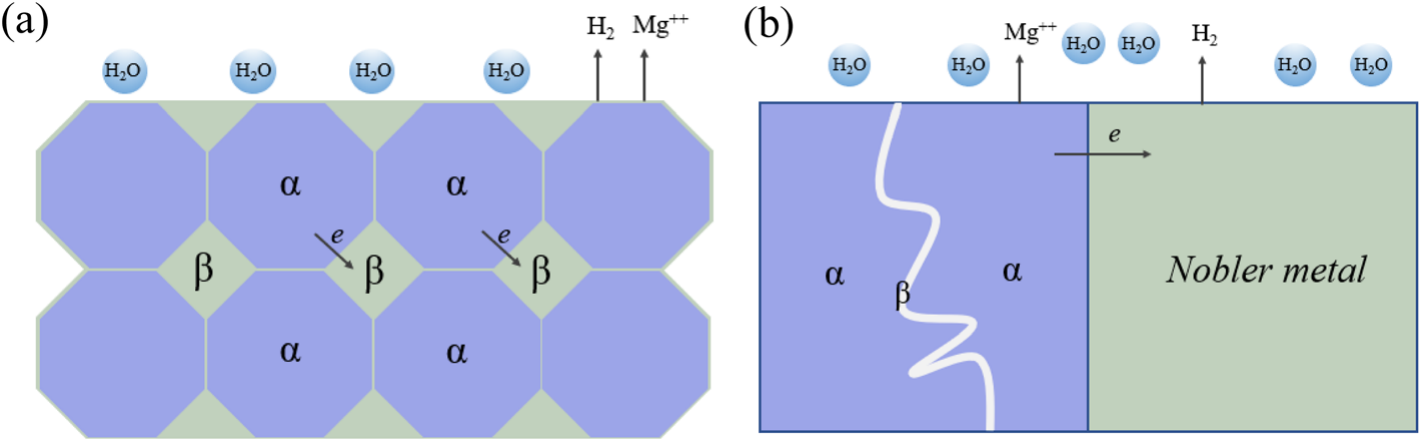
(a) Schematic of internal micro-galvanic corrosion. (b) External galvanic corrosion.^131^

The activity of galvanic reactions can vary by region, particle and phase. Specifically, Mg often acts as a microanode in alloys due to its activity. However, there are many substances that can be microcathode, such as intermetallic compound particles, impurity particles, secondary phase and matrix phase with higher solid solute concentration. Fig. 6 summarizes the microcurrent process and various galvanic corrosion behaviors of Mg alloys. Internal galvanic corrosion or microgalvanic corrosion is due to the presence of impurity grains and cathodic phases on grain boundaries, and the corrosion current flows from α to β, as shown in Fig. 6(a). External galvanic corrosion is caused by contact with noble metal. α grains are pure Mg grains or solid solutions of Mg and its alloying elements (Al, Zn, Ca, Mn and some RE elements). β phase is the second phase formed between grain boundaries and grains.^131^ In general, the electrode potential of β phase is generally higher than that of substrate, which is easy to cause microgalvanic corrosion, so that the surrounding α-Mg phase is preferentially corroded, resulting in serious local corrosion. But this cannot be used as the only judgment of corrosion resistance of β phase. For example, the self-corrosion potential of Mg_2_Ca phase is higher than that of α-Mg phase, but its degradation rate is much higher than that of α-Mg phase. At the same time, anodic β phases with electrode potential lower than α-Mg, such as Mg_24_(Gd, Y)_5_ and Mg_5_(Gd, Y), also exist in Mg alloys.^132^ In addition, the LPSO second phase present in Mg–RE–Zn-based alloys was also found to induce galvanic corrosion in certain alloys, despite this phase having a uniform oxide film and rapid film repair capability. In the as-cast Mg_100−3*x*_(Zn_1_Y_2_)_*x*_ (1% ≤ *x* ≤ 3%, atomic fraction) alloy, the coarse rhombic 18R-LPSO phase located at the grain boundary induces microgalvanic corrosion between grains. With the increase of *x*, the grain size of α-Mg dendrites decreases, the volume fraction of 18R-type LPSO phase increases, and degradation rate accelerates.^133^

#### 3.1.2 Pitting corrosion

**Fig. 7:**
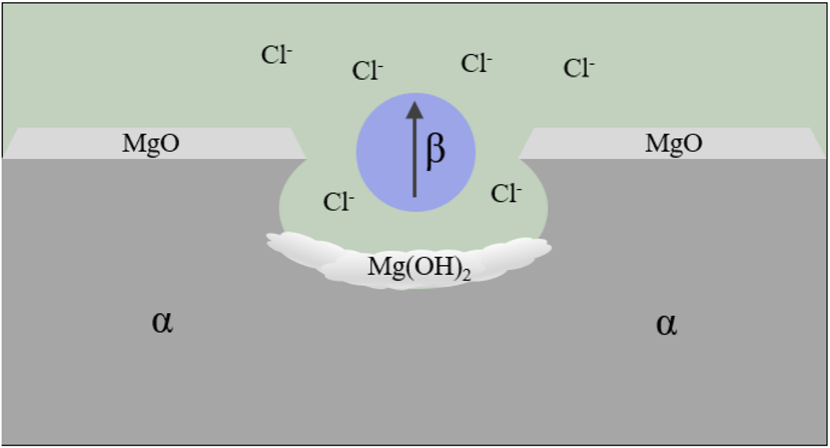
Schematic of pitting corrosion.^131^

Pitting corrosion is dominant. The research of pitting corrosion starts from the microstructure. The alloy structure verified by SEM characterization, electrochemical measurement and local scanning electrochemical technology is usually α-Mg matrix phase + β phase/second phase (*e.g.* Mg_17_Al_12_, AlMn(Fe), Mg_2_Zn, Mg_2_Ca, MgSi_2_, Mg_2_Cu, Al_8_Mn_5_, *etc.*)/intermetallic compounds. As shown in Fig. 7, a typical Mg alloy pitting morphology is shallow pits formed at the rupture of protective passivation layer MgO on the alloy surface after immersion in an aqueous environment with corrosive ions like Cl^−^. The micro-galvanic cells formed in the impurities or the second phase of Mg alloys will continue to accelerate the expansion of corrosion pits until a large number of passivation corrosion products such as Mg(OH)_2_ are precipitated to fill the pits.^131^

**Fig. 8:**
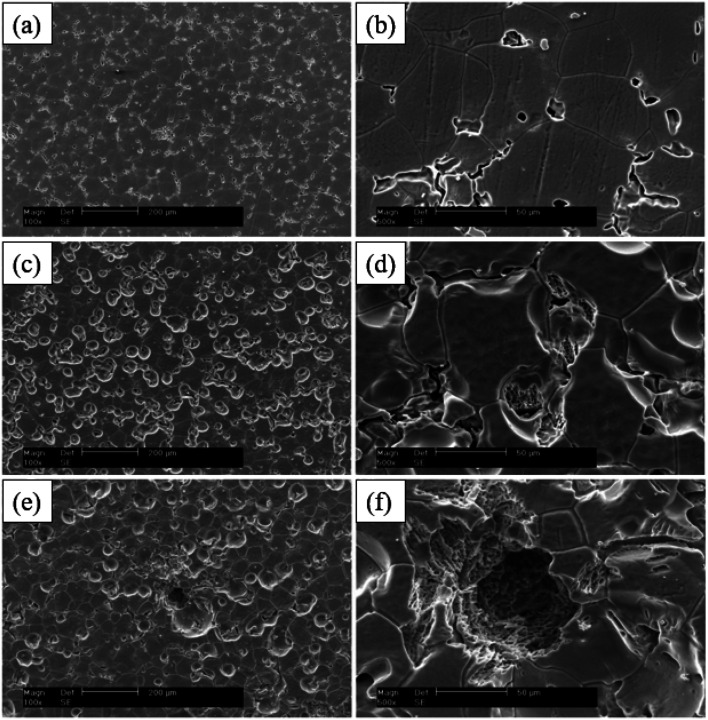
SEM corrosion morphology of cast Mg alloy GW93 immersed in 3.5% NaCl for 30 min (a and b); 48 h (c and d); 72 h (e and f).^110^

It was found that the corrosion pits tended to be in the second phase, because their electrode potential was usually higher than that of the α-Mg substrate.^134^ In the study of RE Mg alloy GW93 (Fig. 8),^110^ researchers found that the pitting corrosion of the alloy could be divided into three stages. In the first stage, the lower potential lead to preferential dissolution of the anodic second phase, which then formed a rare earth oxide film and become inert; in the second stage, corrosion propagated horizontally around the dissolved second phase; in the third stage, corrosion pits were formed and developed deep in the direction of dissolving the second phase, forming pitting corrosion.

Currently, there are two disputes in the pitting corrosion process:

(1) Control steps of pitting corrosion: Williams *et al.* found that the hydrogen evolution rate of the cathode had a strong correlation with the corrosion rate through the corrosion test of AZ31 alloy, so the cathode was judged as a pitting control step.^135^

(2) The pH of anode in pitting zone: one study inferred that the hydrolysis of Mg^2+^ resulted in acidification of the solution by observing the pH value of anode zone to be acidic;^136^ while another study using the scanning vibrating electrode technique found that, according to theoretical calculations, the migration of Cl^−^ accelerated the corrosion in the anodic region, while the hydrolysis of Mg^2+^ did not create an acidic environment.^137^

Pitting corrosion is related to grain size, solution environment, chemical composition and the second phase. Among them: smaller the grain size brings fine grain structure and effectively reduce the number of pitting pits. In AZ31B alloy, it is found that when the grain size is very small, the corrosion morphology of the magnesium alloy develops from pitting corrosion to uniform corrosion;^138^ ion concentration in the solution promote the development of pitting corrosion. For example, the degree of pitting corrosion caused by Cl^−^ usually has a logarithmic relationship with the concentration;^139^ The influence of pH on pitting corrosion is divided into acid and alkali conditions. The general rules currently summarized are: when pH < 0.5, solution is acidic, and the acceleration of pitting corrosion is achieved by dissolving surface film; when 10.5 < pH < 12, the self-corrosion potential and corrosion current are relatively stable, the surface film begins to become stable, and pitting corrosion is inhibited; when pH > 12, the self-corrosion potential rises again, self-corrosion current drops to a very low value, and the protective effect on the surface film is further enhanced.^140^ During corrosion of extruded AM60 at different pH, in acidic and neutral NaCl solutions, the corrosion type of the matrix around the AlMn phase on the alloy surface was pitting corrosion, while the corrosion at the same position in alkaline solution was very uniform and honeycomb-like distribution. The surface of sample at pH 12 was smooth and flat with only a few pits.^111^

#### 3.1.3 Filiform corrosion

The initial process of pitting corrosion and filiform corrosion is the formation of small corrosion pits from Mg matrix. Therefore, these two kinds of corrosion often occur together, but in different propagation paths. The development of pitting corrosion extends into the interior of Mg matrix, and has a lager depth with smaller area. Filiform corrosion is spread on the alloy surface according to a certain path, and the corrosion depth is shallower with a larger area. Compared with Fe and Al alloys, Mg alloys have narrower corrosion wires and slower growth rates.^141^

**Fig. 9:**
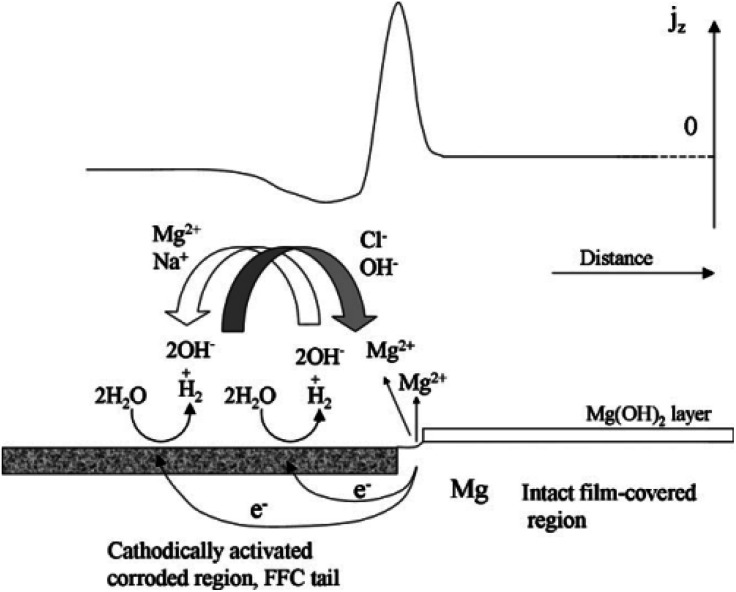
Schematic representation of the mechanism of filiform corrosion under immersion conditions, showing how the regions of the localised corrosion cell correspond with empirically derived SVET *j*_z_*versus* distance profiles.^142^

Taking the corrosion behaviour of pure Mg in salt water as an example, Williams G. *et al.* proposed that the development of corrosion wire is caused by corrosion microbatteries, the head of corrosion wire is anode, while the tail of corrosion wire is cathode. The corrosion wire of cathode is electrically coupled with anode Mg matrix through extension, resulting in a dissolution reaction of anode Mg and the destruction of oxide film. Corrosion product Mg(OH)_2_ of the anode is deposited and lost its activity, and turns into an inert cathode, which can form a new micro-couple with the uncorroded Mg matrix to diffuse filiform corrosion (Fig. 9).^142^

**Fig. 10:**
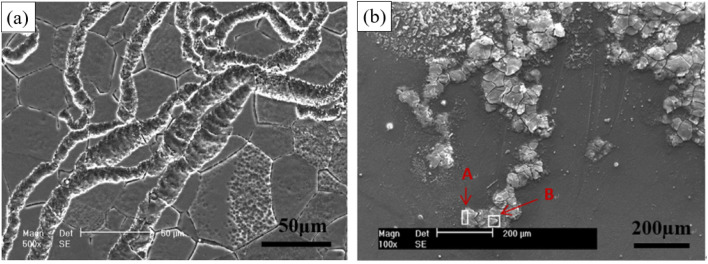
Typical filamentary corrosion morphologies of Mg–3Zn immersed in 0.01 mol per L NaCl solution for 6 h at different scales (50 μm and 200 μm).^121^

Filiform corrosion is closely related to the corrosion medium, microstructure, oxide film and product film. Corrosive medias change the type of corrosion and create conditions for filiform corrosion by forming different surface films. Song *et al.* found that Mg–3Zn alloy exhibited pitting corrosion in Na_2_SO_4_ solution, but filiform corrosion in NaCl solution. When excess Na_2_SO_4_ was added to NaCl, filiform corrosion was transformed into pitting corrosion. The surface film formed in Na_2_SO_4_ solution was denser and more protective than that formed in NaCl solution. The loose corrosion product film formed by Cl^−^ makes it easy to migrate in the film, resulting in non-uniform Cl^−^ dispersion concentration. The high-concentration Cl^−^ accumulation area acts as the filament head, and corrosion tends to extend toward the filament head, and the low-concentration Cl^−^ area left after that forms the filament tail, so the filiform corrosion grows along the horizontal direction. The influence of microstructure on the corrosion development is mainly in the second phase. It was found that the extension direction of corrosion wire is related to the difference between matrix and second phase. In the Mg–8Li alloy, the electrochemical properties of second phase and Mg matrix are less different, and corrosion wire will continue to develop through second phase after the initiation of the boundary. In contrast, the corrosion wires of Mg–Zn–Y–Zr alloys with large differences in electrochemical properties do not pass through the second phase and only stay in Mg matrix.^121,122^ In addition, the development direction of corrosion wire is related to the substrate grain size, orientation, surface oxide film and other factors (Fig. 10).

#### 3.1.4 Stress corrosion cracking

Implants are exposed to complex biochemical and dynamic environment in the human body, and are continuously affected by tensile stress, compressive stress and internal stress. Stress corrosion cracking (SCC) can easily occur under the effects of stress and corrosive internal environment, leading to further complications of sudden fracture.

**Fig. 11:**
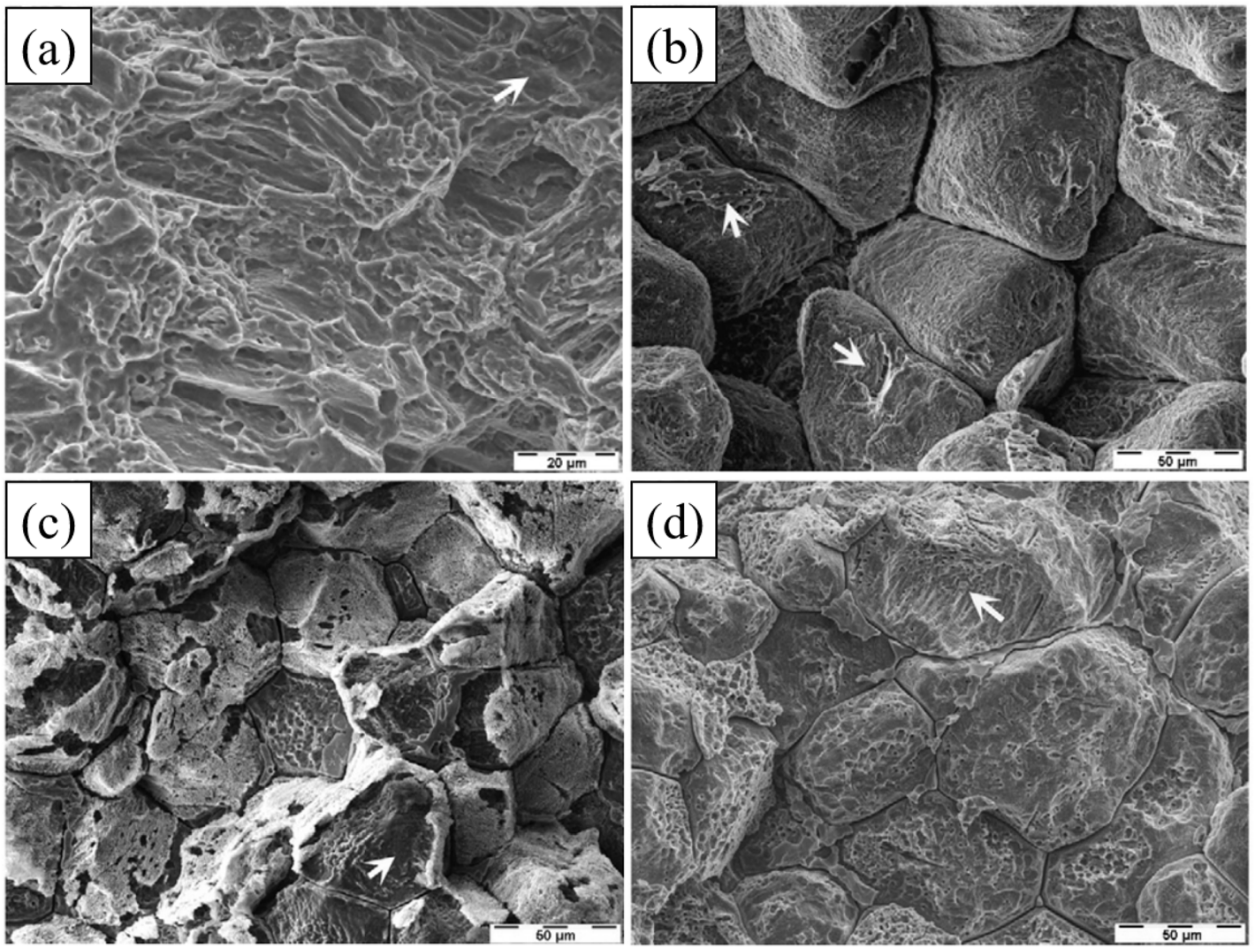
Fracture surfaces of AZ80: (a) transgranular cleavage (arrow) and a few fine dimples; ZE41: (b) predominant IGSCC and isolated TGSCC (arrows) cracking; QE22: (c) IGSCC and TGSCC (arrow); EV31A: (d) IGSCC and TGSCC (arrow).^143^

SCC in Mg alloys can be classified as intergranular (IGSCC) or transgranular (TGSCC). IGSCC is widespread, and current explanations are based on the view that the matrix is continuously dissolved and eventually fractured due to local preferential corrosion. Most second phase has a more positive corrosion potential than the matrix α-Mg and can form galvanic corrosion with matrix. TGSCC is most likely caused by the interaction of hydrogen with microstructure. The hydrogen generated during the reaction between alloy and liquid can easily enter the matrix and undergo hydrogen embrittlement reaction: weakening the bonding of adjacent atoms, reducing the strengthening effect of dislocations and accelerating tip cracking. Fig. 11 below shows the corrosion morphologies of SCC fractures of some Mg–RE alloys and AZ80 in 0.5 wt% NaCl solution at a strain rate of 1.0 × 10^−7^ s^−1^.^143^

Material, tensile stress and environment are the main factors that determine the stress corrosion. Alloying has a significant contribution to SCC resistance, and the effects of Al and Zn on the SCC susceptibility of Mg are very obvious. Catar *et al.* tested the SCC performance of AZ31, AZ61 and AZ91 alloys at different pH. In the air and acid/alkaline environment, the stress corrosion index rank is AZ91 < AZ31 < AZ61, indicating that the effect of Al on the SCC resistance of Mg alloys increases first and then decreases. Mg alloys are most affected by SCC at 6 wt% Al (AZ61).^127^ Therefore, the application of AZ alloys containing Al and Zn should be strictly limited. Al is known to cause various neurological diseases such as dementia or Alzheimer's disease; both Al and Zn are elements that significantly promote SCC. Mg alloys containing these two elements can suppress the promotion of SCC by adding other elements, such as adding Zr or RE to Mg–Zn alloys (ZK60 and ZE10). The influence of environment on SCC is mainly reflected in its Mg(OH)_2_ film. Therefore, the corrosion properties of Mg alloys will have great differences in different pH solutions, and in the stress corrosion test, the effect of pH on Mg alloys is also significant. He *et al.* studied the effect of different pH on the stress corrosion susceptibility of AZ31 the test adopted the method of slow strain rate stretching. In three Na_2_SO_4_ solutions with pH = 2, 7, and 12, it was found that the stress corrosion susceptibility of Mg alloys increases with decrease of pH.^144^ Similarly, the effect of Cl^−^ on SCC is also through the dissolution of the protective film. Temperature, on the other hand, affects SCC by affecting the corrosion rate.

#### 3.1.5 Corrosion fatigue

Based on the corrosion fatigue behavior under complex loading conditions, there are currently several theoretical explanations: (1) pitting corrosion accelerates crack formation; (2) the surface passivation film (protective film) is destroyed under alternating loads and acts as an anode; (3) the precipitated hydrogen is adsorbed on the alloy surface, which weakens the bonding energy strength of the surface, produces hydrogen embrittlement, and accelerates the initiation of corrosion fatigue cracks. (4) The damage evolution model driven by coupling of microstructure and corrosion morphology. (5) The degree of intrusion/extrusion and retardation of oxide film on cyclic slip (competitive relationship). Among them, pitting-induced corrosion fatigue has been widely reported.^145^

**Fig. 12:**
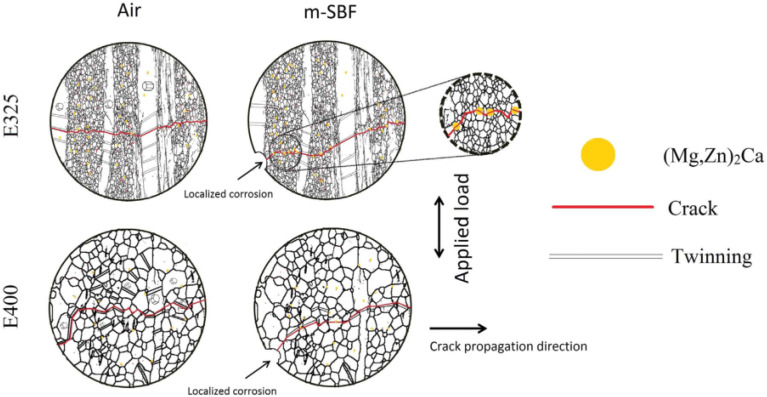
Schematic diagram of fatigue cracking of ZX10 extruded at 325 °C and 400 °C when tested in air and m-SBF.^126^

The pitting pits formed during corrosion process accelerate the generation of fatigue cracks, especially in the body fluid environment rich in Cl^−^, the cracks are easy to expand to a critical state, and fracture or failure occur. Gu *et al.* conducted corrosion fatigue tests on AZ91D and WE43 alloys in SBF solution, and found that the initial cracks of both alloys originated from corrosion pits.^123^ As shown in Fig. 12, Jafari *et al.* also found that the initial crack source was corrosion pit when ZX10 was subjected to corrosion fatigue test in m-SBF solution, and the anodic dissolution of noble intermetallic phases (Mg, Zn)_2_Ca accelerated short fatigue crack growth by providing a more convenient path for crack growth.^126^ This theory is usually used to explain the phenomenon of localized corrosion; however, it cannot explain the corrosion fatigue phenomenon that occurs in the absence of corrosion pits on the surface of the alloy.

The factors affecting corrosion fatigue cracks and their propagation can be classified as follows:

(1) Material properties, including alloy composition and processing technology (improving grain size and secondary phases); for example, alloying, high purification, surface coating, processing technology such as forging or aging treatment are used to improve the corrosion performance of the alloy.^146^

(2) External factors, including mechanical factors (maximum fatigue load, stress, stress ratio, loading frequency) and corrosive factors (corrosive medium, temperature, *etc.*). Implants experience complex multiaxial loads in the human body, including tension, compression, bending and torsion. The loading frequency and amplitude are the most significant mechanical indicators of corrosion fatigue properties. Zeng *et al.* found that the fatigue life of extruded AZ61 decreased with the decrease of loading frequency at 1–10 Hz in air. This is because at low frequency, the oxygen in the air has enough time to react with the matrix to generate oxidation products, resulting in irreversible plastic deformation during corrosion fatigue. And above 10 Hz, the fatigue life is independent of frequency.^147^ When loading frequency is constant, the stress amplitude also has an influence on the fatigue test results. In the high-cycle rotational bending fatigue test of AZ80, Shiozawa *et al.* pointed out that the irreversibility of twinning deformation could be used to control the initiation of fatigue cracks under high stress amplitude, and at low stress amplitudes, the initiation of fatigue cracks could be controlled by adjusting the slip deformation mechanism.^124^ In a study on corrosion fatigue of AZ80-T5, Bhuiyan *et al.* showed that under high stress amplitude, crack nucleation was formed by the growth of a single pit to a critical size; at low stress amplitude, the surface of the sample appeared multiple etch pits grow and coalesce to form one large pit. According to the previous discussion, pits are the main cracking source of corrosion fatigue, and the application of stress has an important effect on pit formation, nucleation and crack nucleation.^148^ Temperature and humidity also affect fatigue crack growth. Generally, when the relative humidity is less than 60%, it has no effect on the fatigue strength. When the relative humidity is larger than 90%, the fatigue strength is significantly reduced.^149^

### 3.2 Dissolution of magnesium alloys in body fluids

Due to the complexity in body fluid environment (anions, glucose, protein, flow rate, microorganisms, *etc.*), the degradation behavior of alloys in body fluids is diverse. *In vivo* testing has high cost and ethical burden, most studies on the effects of body fluids on Mg corrosion have been conducted through *in vitro* simulations. Simulated body fluids containing different types and amounts of ionic and organic components create sufficient variable conditions for corrosion tests. From Table 4, there are significant differences in the composition and ion concentration of these simulated body fluids.

**Table 4:** Comparison of the composition of plasma and several commonly used *in vitro* simulated body fluids^150^^a^

Component	Units	Plasma	0.9% NaCl	PBS	HBSS	D-HBSS	Ringer's solution	EBSS	MEM	DMEM	SBF	SBF-T	SBF-H	SBF-L
Na^+^		142	154	146	141.6	141.6	113.6	144	143	127.3	142	142	142	142
K^+^		5	—	4.1	5.81	5.81	1.88	5.4	5.4	5.4	5	5	5	5
Mg^2+^		1.5	—	—	0.81	—	—	0.4	0.4	0.8	1.5	1.5	1.5	1.5
Ca^2+^	mmol dm^−3^	2.5	—	—	1.26	—	1.08	1.8	1.8	1.8	2.5	2.5	2.5	2.5
HPO_4_^2−^		1	—	9.5	0.78	0.78	—	1	0.9	0.9	1	1	1	1
HCO_3_^−^		27	—	—	4.065	4.065	2.38	26	26	44.1	4.2	27	27	27
Cl^−^		103	154	140.6	144.8	143	1153	125	125	90.8	147.8	125	103	103
SO_4_^2−^		0.5	—	—	0.81	0.81	—	0.4	0.4	0.8	0.5	0.5	0.5	0.5
Protein		35–80	—	—	—	—	—	—	—	—	—	—	—	—
Glucose	g dm^−3^	0.9–1.1	—	—	1.0	1.0	1.0	1.0	1.0	4.5	—	—	—	—
Amino acids		0.25–0.40	—	—	—	—	—	—	0.95	1.6	—	—	—	—

Vitamins		Unknown	—	—	—	—	—	—	0.008	—	—	—	—	—
Buffer		—	—	—	—	—	—	—	—	Tris–HCl	HEPES	Tris–HCl	HEPES	—

a PBS: phosphate-buffered saline; HBSS: Hank's balanced salt solution; D–HBSS: Dulbecco's–Hank's balanced salt solution; EBSS: Earle's balanced salt solution; MEM: minimum essential medium; DMEM: Dulbecco's modified Eagle's medium; SBF: simulated body fluid; SBF-T: 50 mM Tris-27 mM HCO_3_^−^; SBF-H: 50 mM HEPES-27 mM HCO_3_^−^; SBF-L: 22 mM Na-L4actate-27 mM HCO_3_^−^.

#### 3.2.1 Effects of inorganic salt ions

In corrosive environments containing chlorine solutions, such as 0.9% NaCl (physiological saline), 3.5% NaCl (simulated sea water), the oxide film of Mg alloy is easily attacked by Cl^−^ and serious corrosion occurs. Cl^−^ has small volume, fast moving speed, low degree of hydration and strong penetrating power to oxide film, which make it easy to cause continuous corrosion (usually pitting corrosion). Similarly, in simulated human body fluids, increased Cl^−^ concentration accelerates the degradation of the alloy.^151^ Taltavull *et al.* studied the effect of Cl^−^ concentration (0 mol dm^−3^, 1 mol dm^−3^) on the corrosion behavior of pure Mg, AZ91 and ZE41 in modified HBSS. The increase of Cl^−^ induced localized corrosion in all tested materials, among them, the local corrosion of AZ91 and ZE41 containing the second phase was more serious.^152^ SO_4_^2−^, NO_3_^−^ and Br^−^ can also be adsorbed on the surface of Mg(OH)_2_ film, changing the electric field distribution of adsorbed electric double layer on the alloy surface and accelerating the corrosion.^153^

When there is no moisture in the gas, fluorine, chlorine, bromine and iodine have no corrosive effect at not too high or normal temperature. When the gas contains a small amount of water (below the dew point), fluorine is still non-corrosive, bromine is corrosive to a certain extent, and wet iodine has serious corrosion to Mg alloys. Chloride salt has strong penetrating ability and can be deposited on the alloy surface pattern, reducing the relative humidity of water condensation, thereby accelerating electrochemical corrosion.^154^

HCO_3_^−^ is anion that provides buffering in simulated fluids. HCO_3_^−^ accelerates corrosion of AZ91 in the early stage of immersion, and later due to precipitation of MgCO_3_ and CaCO_3_, the passivation alloy surface formed and slowed down the corrosion rate and localized corrosion is suppressed.^155^

Liang *et al.* studied the effect of different anions on AZ80, which exhibited localized corrosion in solutions containing Cl^−^, HCO_3_^−^, and SO_4_^2−^ during short-term immersion. After adding HPO_4_^2−^, AZ80 exhibited uniform corrosion. As the soaking time increases, the corrosion rate decreased due to the reduction of active surface. Among these anions, SO_4_^2−^ had good nucleophilicity similar to Cl^−^, which could be easily adsorbed on thin film and had the most obvious corrosion promoting effect. HPO_4_^2−^ prevents pitting corrosion and surface passivation by forming Mg precipitation which can inhibit the corrosion effect of SO_4_^2−^ to a certain extent.^156^

Inorganic salt cations may also affect the corrosion behavior in simulated fluids. Song *et al.* reported that phosphate ions usually combine with Mg^2+^ and Ca^2+^ to form a (Mg, Ca)–P layer in HBSS solution, which can significantly inhibit corrosion, and this corrosion layer was also found in *in vivo* studies.^157^ The same CaCO_3_ precipitation can also inhibit magnesium corrosion. Marco *et al.* studied the corrosion behaviors in D-HBSS solution and *in vivo*, and found that due to the lack of Ca^2+^, the corrosion layer formed was significantly different from that *in vivo* samples. Therefore, Ca^2+^ is crucial to the formation of the corrosion product layer, which in turn affects the corrosion mechanism of Mg implants *in vitro* and *in vivo*.^158^

#### 3.2.2 Effects of organic ingredients

Although the simulated fluids used in most *in vitro* studies do not contain organic components, actual plasma contains a considerable amount of organic components, such as proteins, amino acids, vitamins and glucose.

##### 3.2.2.1 Effects of protein and amino acids

**Fig. 13:**
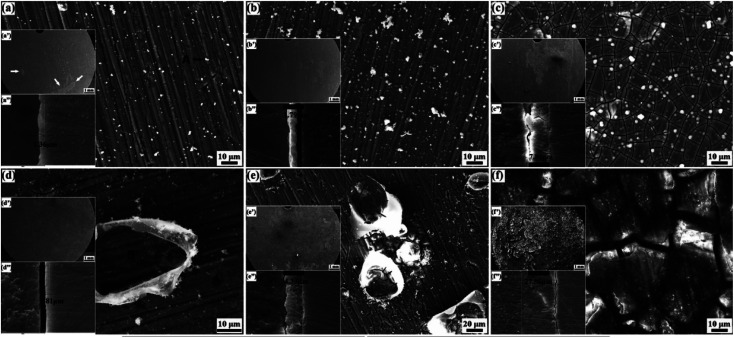
Surface morphologies of ZK21–0.2Sc alloy after immersion: (a–c) Hank's, (d–f) Hank's + BSA; (a and d) 6 h, (b and e) 12 h, (c and f) 168 h.^163^

Proteins, especially albumin, are essential substances in human plasma. Corrosion of Mg alloys by biomacromolecular proteins needs to be further considered. Proteins mainly participate in and affect the degradation through adsorption and chelation, and their molecules bind to the alloy surface through electrostatic interactions of bonds to form a layered structure.^159^ These processes are affected by the alloy properties, the composition of the solution and protein concentration. The effect of protein adsorption on the degradation has both promoting and inhibiting effects. When the protein layer adsorbed on the alloy surface plays a protective role, the corrosion process is inhibited. Furthermore, inhibitory effect is proportional to albumin concentration; when certain groups in the protein (such as COOH^−^) release ions and change the pH of solution, instead, the corrosion process is promoted.^160^ Zhang *et al.* found that the corrosion rate of Mg–Nd–Zn–Zr alloy decreased slightly after adding 10% protein to SBF. This may be due to the protein adsorbing on the implant surface, which acts as a corrosion shield to reduce the corrosion rate of implants.^161^ However, Harandi *et al.* found that bovine serum albumin adsorbed on the implant surface and played a protective role only in the first few hours. As the immersion time increases, the protein and corrosion products formed chelates, and the protective film of alloy become invalid.^162^ Li *et al.* used albumin-containing hank solution to soak Mg–1.5Zn–0.6Zr–0.2Sc (ZK21–0.2Sc), and came to a similar conclusion that the corrosion rate of alloy first decreased with the increase of protein concentration, but this protective effect lasted only six hours. As shown in Fig. 13(a–c), the corrosion continued to increase after 6 h in Hank's solution, and the thickness of the deposited corrosion film also increased. In contrast, Fig. 13(d–f) are alloys in Hank's + BSA with many albumin crystals attached. The corrosion product is a passivated translucent film. Corrosion below the attached albumin crystals in panel (d) is relatively weak, indicating the corrosion protection in the early stage of albumin. This was followed by more severe corrosion than in Hank's solution as albumin aggregated and decomposed.^163^

Although the concentration of amino acids in plasma is much lower than that of proteins, the corrosion mechanism of amino acids on Mg alloys is still unclear due to their wide variety and synergistic mechanism with other components of plasma, and amino acids are also present in some simulated body fluids such as MEM and DMEM. Yamamoto *et al.* used an EMEM solution containing amino acids to immerse pure Mg and found that amino acids reduced the barrier effect of the insoluble salt layer on Mg dissolution, thereby accelerating the corrosion of pure Mg.^164^ However, Wang *et al.* studied the synergistic effect of amino acid (l-cysteine) and glucose on the corrosion of pure Mg and found that l-cysteine alone could inhibit the corrosion of Cl^−^ on pure Mg. The functional group of Schiff base formed by the combination of amino acid and glucose inhibits the formation of insoluble salt barrier and accelerates the corrosion process.^165^ In addition to the synergistic effect, the effect of amino acid concentration on the corrosion rate of magnesium alloys is also complex, and the type of amino acid and corrosion environment may change the effect of concentration on corrosion. In general, low concentrations of amino acids may have a protective effect, while high concentrations may accelerate the rate of corrosion.^166^ In a study, the corrosion current IE of CP Mg and Mg–0.5Ca was tested using cysteine at three concentrations of 10^−2^, 10^−3^ and 10^−4^ M. In CP Mg, it was observed that low concentration of cysteine had an inhibitory effect on corrosion. As concentration increased, the IE of both Mgs increased significantly. However, in CP Mg, the IE first increased and then decreased with the increase of concentration. In the test of other types of amino acids, it was found that they can basically accelerate the corrosion of Mg–0.8Ca, but half of them (13/24) also showed a high-concentration inhibitory effect on CP Mg similar to the above.^167^ Therefore, it is more common for the increase of amino acid within a certain concentration range to accelerate the corrosion of magnesium alloys.

##### 3.2.2.2 Effects of glucose

**Fig. 14:**
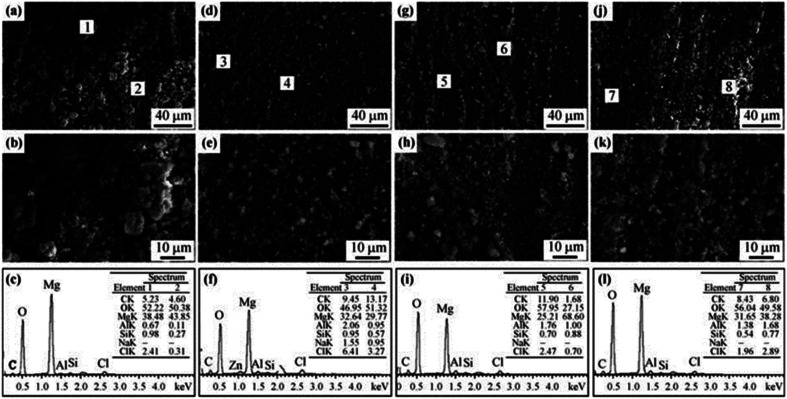
SEM and EDS analyses of AZ31 alloy after immersion in 0.9% NaCl and 6.118 g per L Tris solutions with different glucose contents for 18 h: (a–c) 0 g per L glucose; (d–f) 1 g per L glucose; (g–i) 2 g per L glucose; (j–l) 3 g per L glucose.^169^

In patients with diabetes, the effect of glucose levels on the degradation cannot be ignored. The corrosion effect of glucose mainly depends on its conversion to gluconic acid. Glucose protects Mg alloy samples, while gluconic acid destroys them. Cui *et al.* found that the corrosion rates of pure Mg matrix and Mg–1.35Ca samples increased with the increase of glucose concentration in simulated body fluid saline.^168^ The team then studied the corrosion behavior of AZ31 in unbuffered saline solution and Tris salt solution, and found that low concentration of glucose could inhibit the corrosion of magnesium alloys. Low concentrations (1.0 g L^−1^) of glucose complexed with Mg^2+^ ions and hindered the further erosion of Cl^−^ ions; while high concentrations (2.0 and 3.0 g L^−1^) of glucose were converted into gluconic acid, which promoted the adsorption of Cl^−^ on the sample surface. The hydrolysis of amino groups in Tris increases the initial pH of the salt solution, promotes the conversion process of glucose to gluconic acid, and reduces the corrosion resistance of magnesium alloys.^169^ The corrosion morphology caused by glucose is shown in Fig. 14. The corrosion of solutions containing low concentrations of glucose (Fig. 14(d) and (e)) was more uniform and flatter than the other three groups, showing the protective effect of glucose. The two groups of high-concentration glucose solutions formed wider and deeper crack fracture corrosion products. The element content of EDS showed that the corrosion products are mainly Mg(OH)_2_. In above studies, the type of solution also had a significant effect on the reaction between glucose and substrate. In physiological saline solution, gluconic acid destroyed the protective magnesium hydroxide corrosion product film, promoted Cl^−^ ions to the surface of the sample, and accelerated the corrosion; while in Hank's solution, gluconic acid could chelate Ca^2+^ in the solution, ions accumulated on the surface of the sample, promoted the deposition of calcium and phosphorus products, and reduced the degradation rate.

#### 3.2.3 Effects of experimental parameters

##### 3.2.3.1 Effect of pH

**Fig. 15:**
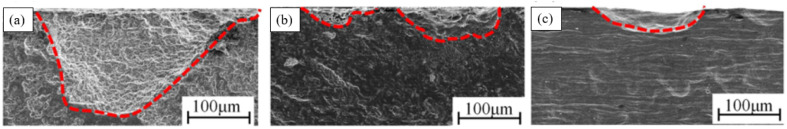
Pits of ZK60 on the fractures in three solutions. (a) pH 5.2, (b) pH 7.4 and (c) pH 9.0.^176^

Changes in pH may significantly affect the corrosion of Mg alloys. At low pH (acidic conditions), the corrosion rate increases due to hydrogen evolution reactions at the material surface. This reaction leads to the formation of hydrogen bubbles on the surface of the alloy, which causes pitting corrosion and accelerates the corrosion rate.^170^ In addition, low pH conditions also lead to the dissolution of the protective oxide layer formed on the surface of the alloy, further accelerating the corrosion process.^171^ As the pH value decreases, the corrosion potential moves to a more negative (more active) direction, which is the same law as the increase of chloride ion concentration on corrosion. However, at pH 2, AZ63 reached the maximum corrosion rate, and the change of chloride ion concentration did not significantly affect the corrosion potential.^172^ On the other hand, at high pH values (alkaline conditions), the corrosion rate of Mg alloys can decrease due to the formation of a passive film on the surface of the material. This film is a complex oxide that provides a protective barrier against further corrosion. However, if the pH is too high, the passive film may dissolve, leading to an increase in the corrosion rate.^173^ Li *et al.* explained the corrosion behavior of AZ31B in a highly alkaline environment: at pH ≥ 13, although a thin and thick oxide film is formed on the substrate, Al–Mn intermetallic compounds undergo dealloying dissolution at high pH values, the passivation film at the oxide interface between the primary α-matrix and the eutectic α phase is broken down; a uniform and stable passivation film is formed when the pH is 12; when the pH is between 10–11, the corrosion mainly occurs in the primary α-matrix.^174^ The main corrosion product of magnesium in neutral and alkaline solutions is Mg(OH)_2_, however, in neutral solutions, Mg(OH)_2_ is formed by dissolution–precipitation reactions; while in alkaline solutions, formed by solid anode reaction. The Mg(OH)_2_ surface film formed under neutral conditions is less tolerant and cannot exist stably when the pH value is lower than 10.5.^175^ The pH of human blood is about 7.40, and the dissolution of the Mg(OH)_2_ corrosion layer will lead to accelerated corrosion of implants. In simulated body fluids in an unbuffered system, the pH of the solution is usually higher than the normal physiological pH of human body when corrosion occurs. The solubility of the corrosion layer decreases with the increase of pH, and the protective effect also increases. Sun *et al.* performed cyclic sine wave experiments of corrosion and fatigue crack growth of ZK60 in PBS at pH values of 5.2, 7.4 and 9.0, and found that with the increase of pH, the integrity and density of the protective layer increased. Over time, the protective layer had an obvious and then weak inhibitory effect on pitting corrosion and crack propagation.^176^ As shown in Fig. 15, pitting corrosion is the main corrosion mechanism in pH 5.2 solution, and filiform corrosion exists in pH 7.4 solution. Corrosion is shallower and more uniform in pH 9.0 solutions. The depths of corrosion pits for these types of corrosion decrease sequentially.

##### 3.2.3.2 Effect of temperature

Temperature changes the corrosion resistance properties by altering their chemical reactivity as well as microstructure. Therefore, the conclusions drawn from the corrosion tests carried out at room temperature may be in error with the situation at the actual body temperature (37 °C). Temperature is positively correlated with corrosion because it brings more activation energy. Kirkland *et al.* found that the corrosion rates of CP–Mg, Mg–0.8Ca and Mg–lZn increased significantly when the immersion temperature of the HBSS solution was increased from 20 °C to 37 °C.^177^ Also, the AZ alloys have the characteristic of negative shift of the self-corrosion candle potential with the increase of the ambient temperature, resulting in an increase in the corrosion rate.

##### 3.2.3.3 Influence of flow field environment

**Fig. 16:**
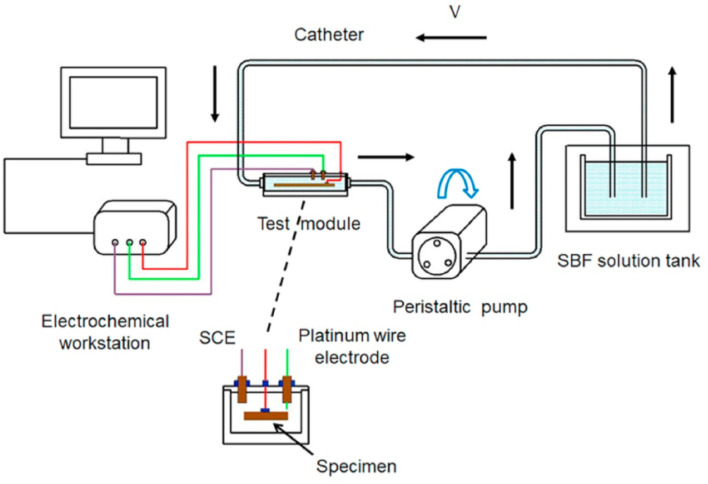
Schematic diagram of a dynamic flow field test device.^181^

In blood vessels, the blood flow velocity varies between 0.1 and 1.0 m s^−1^, depending on the diameter of vessel. With the advancement of experimental conditions, the effect of blood flow on degradation is being extensively studied. It has been widely recognized that the degradation rate of Mg alloys in flowing state is significantly higher than that in static state. The flow field can change the corrosion rate through the action of shear force and mass transfer process.^178^ Corrosion at low flow rates is mainly controlled by the mass transfer process. Shear force at high flow rates will destroy the surface layer to form erosion corrosion, but there is no precise critical flow rate to define high and low flow rates. Levesque *et al.* conducted a simulation of the degradation in cardiovascular systems and found that the corrosion rate and corrosion mechanism of AM60B varied with the flow rate. Under the action of low shear stress of AM60B, the surface of the sample was uniformly corroded; when in high the shear stress, uniform corrosion and local corrosion occurred at the same time.^179^ If there is no effective corrosion product protection, the acceleration effect of the flow field on corrosion will be more obvious. Mirco Peron *et al.* used simulated body fluid to flush AZ31 and found that its corrosion rate increased with the increase of liquid flow rate, and the corrosion continued to accelerate with time until the sample was basically degraded. The main reason is that the surface of the sample is uneven, and the shear force caused by the flow field scouring the surface is larger. The oxide layer of the surface degradation product can be taken away in time and the local pH can be balanced, so that the corrosion rate can be maintained. However, the change of surface topography brought about by the flow field reduces the probability of perforation of the sample due to localized corrosion.^180^ The flow field environments are more difficult to form pitting pits than on the static environment alloy surface. The reason could be: (1) the flow medium is easier to transport oxygen to the metal surface, which promotes the formation of oxide passivation film. (2) The flow field can wash away deposits and protective film layers on the metal surface, making the surface smoother and creating conditions for uniform corrosion. In the above studies, it was also found that the temperature can act together with the flow field to affect corrosion, and the corrosion rate is proportional to the temperature of the flow field. Also, the orientation of the sample changes the corrosion resistance in the flow field. Zhu *et al.* tested AZ80 specimens with three orientations of ND, ND45 and TD. The specimens were tested in static (TD > ND45 > ND) and dynamic (TD > ND) environments and showed anisotropic corrosion performance which is related to the peak intensities of the crystal planes with different orientations. From the dynamic test device in Fig. 16, the sample size is 10 mm × 9 × 10 mm, the pH of the SBF solution was 7.4 and the temperature was maintained at 37 ± 0.5 °C. Three flow rates of 0, 1 and 1.5 ml s^−1^ can be achieved by adjusting the power of the peristaltic pump.^181^

#### 3.2.4 Corrosion rate difference of magnesium alloy *in vitro* and *in vivo*

The evaluation of the difference in the corrosion rate of Mg alloys *in vivo* and *in vitro* cannot be accurate and uniform, because the experimental conditions and corrosion rate evaluation methods are diverse. *In vitro* corrosion tests are designed to replicate the specific environmental conditions to which a material may be exposed *in vivo*, but they may not fully capture the complexity and variability of the *in vivo* environment. In addition, *in vivo* corrosion rates may also vary depending on the specific implant site. *In vitro* corrosion rate evaluation methods include immersion test and electrochemical test, *etc.*, while *in vivo* tests include weight loss method, μCT, *etc.*^182^ At the same time, it is very difficult to establish the correlation between *in vitro* and *in vivo* corrosion rates under the existing experimental conditions.^183^

Although the evaluation methods are multivariate and complex, in the reported data, the overall corrosion rate of Mg alloys *in vitro* is generally higher than that obtained *in vivo*, and the corrosion factors are similar when considering some media used for *in vitro* degradation evaluation.^184^ Waizy *et al.* reported the difference in *in vivo* and *in vitro* corrosion of a series of Mg alloys. Corrosion conditions *in vitro* and *in vivo* were controlled. For AZ31, the average corrosion rate *in vivo* is 0.3 mm year^−1^ (weight loss, rat subcutaneous), and the average corrosion rate *in vitro* is 0.85 mm year^−1^ (immersion, EBSS/MEM/MEM + BSA); for Mg1Zn, the average corrosion rate *in vivo* and *in vitro* is 0.26 mm year^−1^ and 0.96 mm year^−1^ respectively; for Mg–Mn, the average corrosion rate *in vivo* and *in vitro* is 0.298 mm year^−1^ and 1.19 mm year^−1^ respectively; for Mg–1.34Ca–3Zn, the average corrosion rates *in vivo* and *in vitro* are 0.92 mm year^−1^ and 4.47 mm year^−1^ respectively.^185^ Another study tested AZ91 under similar conditions, the average corrosion rate *in vivo* (weight loss, rat intramuscular) was 0.56 mm year^−1^, and the average corrosion rate *in vitro* (Notr's solution) was 6.23 mm year^−1^.^186^ The surface treatment significantly improves the corrosion resistance, and the average corrosion rate of PCL coating AZ91 *in vivo* is reduced to below 0.01 mm year^−1^, and the average corrosion rate *in vitro* is reduced to about 0.2 mm year^−1^.^187^

*In vivo* and *in vitro* corrosion rates vary in magnitude. Generally, the *in vivo* corrosion rate is low, and some are even lower by several orders of magnitude. The reasons may be as follows: (1) the tissue environment in the body is complex, and there may be errors in the existing calculation method of corrosion rate; (2) the temperature used to determine the corrosion rate exceeds the physiological range; (3) the corrosion medium and buffer system of the corrosion solution tested *in vitro* is quite different from the physiological environment;^188^ (4) as discussed in Section 3.2.2, the organic components in the physiological environment, such as protein adsorption and chelation, the type and concentration of amino acids, *etc.*, will change the corrosion behaviour; (5) dynamic corrosion in physiological environment: when the concentration of ions in the surrounding fluid reaches equilibrium, the corrosion of Mg alloys will stop. The electrolyte concentration in the organism is in a steady state and cannot meet the chemical equilibrium conditions, which may have a continuous impact on the corrosion.^189^

## 4. Alloying

### 4.1 Elements selection

Solution and grain refinement strengthening are the main strengthening methods in Mg alloying. Therefore, the elements with better solid solution with Mg should be given priority during elements selection. According to the Hume-Rothery solid solubility criterion, when the radius difference between the solvent atom and the solute atom exceeds 15%, it is not conducive to the formation of a solid solution, and the solid solubility will be very small. With the application of rapid solidification technique, the difference in radius between atoms can be increased to 30%, alloying of more elements becomes available.^190^ The atomic radius of Mg is 0.160 nm. Among the metal elements, K, Rb, Cs, Sc, Ba, *etc.* are not suitable for solid solution strengthening due to their large radius, which is more than 30% different. Similarly, elements Cr, Fe, Co, Ni, Cu, *etc.* are not suitable because their radius is smaller than that of Mg by more than 30%. Also, chemical affinity affects the strengthening mechanism. The bonds between atoms with an electronegativity difference of less than 0.5 are non-polar covalent bonds, which are conducive to the formation of solid solutions. The electronegativity of Mg (1.2) is quite different from the electronegativity of metal elements K, Rb, Cs, Fe, Co, Ni, Cu, Mo, Tc, Ge, Sn, Sb, *etc.*, which is not conducive to the formation of solid solutions. Since the implanted metal needs to be completely degraded eventually, from the viewpoint of degradability, the metal elements Ti, Ge, Zr, Tc, Ru, Os, Rh, Ir, Pt, Pd, Cu, Ag and Au are not suitable for biodegradation; considering biocompatibility, Be, Ga, Cd, V, Nb, Ta, Cr, Co, Ni, Pb and Ti *etc.* are excluded.

**Fig. 17:**
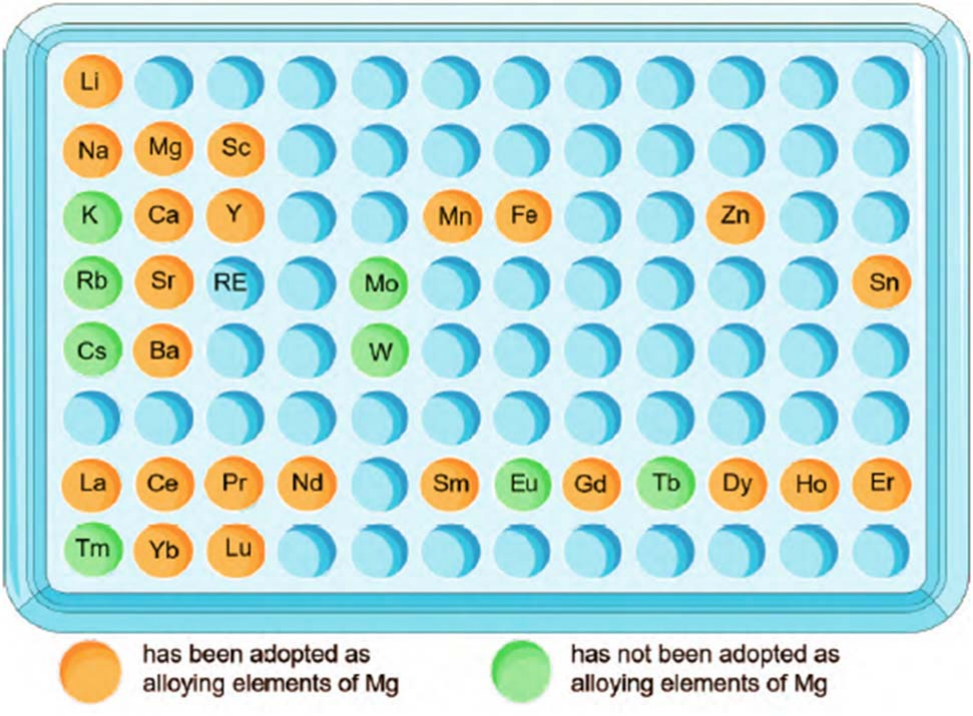
Suitable elements for magnesium alloying and their applications.^191–193^

After the above screening process, Li, Na, Zn, Sn, Sc, Ca, Y, Mn, Sr, Ba and Re are finally thought to be suitable as candidate elements for magnesium alloying (Fig. 17).

The addition of alloying elements can change the composition and structure of the second phase, as well as their morphology and distribution. The following will be classified and discussed according to the characteristics of alloying elements affecting the microstructure, mechanical properties, corrosion resistance and biocompatibility of Mg alloys.

### 4.2 Biofunctional elements

(1) Ca: Ca can significantly improve the oxidation resistance of Mg alloys at high temperatures, and the equilibrium partition coefficient of Ca in Mg is less than 1, which can refine the structure and inhibit degradation. The newly formed Mg_2_Ca phase is the key to control the microstructure and strength. However, there are also reported examples of microgalvanic corrosion induced by the second phase.^194^ On the outer surfaces of pure Mg, AZ31, AZ80 and AZ91D, Ca element enriches as an impurity and forms CaO thin films. Ca in Mg–Li alloy plays a better protective role by promoting the formation of Ca(OH)_2_, CaCO_3_, Ca_3_(PO_4_)_2_ surface film, and increasing the density of Mg(OH)_2_ outer layer^195^

**Fig. 18:**
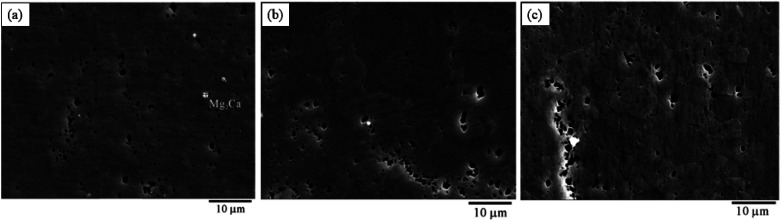
SEM images of the (a) Mg–0.54Ca alloy; (b) Mg–0.79Ca alloy; (c) Mg–1.35Ca alloy.^198^

The solubility limit of Ca in Mg is 1.34 wt%, in general, the corrosion resistance of alloys increases with the increase of Ca content. Harandi *et al.* found that the corrosion rate of Mg–*x*Ca (*x* = 0.7, 1, 2, 3, 4) increased uniformly with increasing calcium content.^196^ However, Zeng *et al.* found that the highest hardness, ultimate tensile, yield strength and corrosion resistance of Mg–0.79Ca were higher than those of Mg–0.54Ca and Mg–1.35Ca. This situation is attributed to the homogeneity of the microstructure. The contribution of Ca to corrosion resistance increases first and then decreases, and they believe that there are two sides: (1) the introduction of Ca leads to grain size refinement, which improves corrosion resistance. (2) Ca also leads to an increase in the volume fraction of the Mg_2_Ca phase (as shown in Fig. 18(c), there are obvious Mg_2_Ca phases), which can form galvanic corrosion with the α-Mg matrix to corrode the alloy.^197^ With the increase of Ca, the effect of the formation of the second phase on corrosion will be more obvious. A series of Mg–*x*Ca (*x* = 0.5, 1.25, 2.5, 5, 10, wt%) were tested, and it was found that with the increase of Ca, the Mg_2_Ca second phase distributed at the grain boundary would also increase, and Mg–10Ca showed the fastest degradation.^198^

(2) Sr: Sr is a trace element in the body, and 99% of it exists in bones. Sr has an osteoinductive effect to help induce the formation of osteoblasts and promote the rapid integration of implants with bones. The Sr-containing drug strontium ranelate (SR) has been used to treat osteoporosis. The addition of Sr can refine the grains in two ways. On one hand, Sr increases the degree of supercooling before the solid–liquid interface, thereby promoting the nucleation of the α-Mg phase; on the other hand, the solid solubility of Sr in Mg is low, only 0.11 wt%, which easily leads to the segregation of Sr. The excess Sr will accumulate at the solid/liquid interface and may damage the grain surface or change the direction of grain growth. Therefore, the grain growth becomes slow and the grains are refined.^199^ The improvement of mechanical properties after fine grains and biocompatibility brought about by Sr are remarkable. Gu *et al.* studied a series of hot-rolled degradable Mg–(1–4 wt%)Sr binary alloys, and the study showed that the Mg–2Sr hot-rolled alloy had the lowest corrosion resistance and the highest strength. *In vitro* and *in vivo* experiments show that the Mg–2Sr has good biocompatibility. Implantation experiments in mice showed that Mg–2Sr promoted new bone formation and bone mineralization without any side effects.^200^ Another study showed that the as-cast Mg–0.5Sr had the lowest degradation rate in simulated body fluids, and its leaching solution had no toxicity on human vascular endothelial cells. Three-week animal implantation experiments show that Mg–0.5S alloy does not cause any harm to animals.^201^

### 4.3 Biocompatible elements

**Fig. 19:**
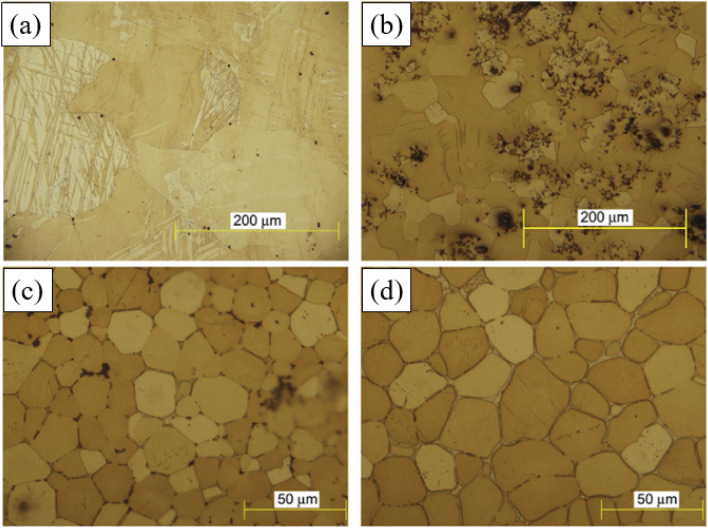
Refinement effect of Zr and Sr on magnesium alloys (a) Mg; (b) Mg–5Zr; (c) Mg–5Zr–2Sr; (d) Mg–5Zr–5Sr.^204^

(1) Zr: Zr has low *in vitro* toxicity and good vivo biocompatibility, degradability, non-mutagenicity and carcinogenicity. In blood compatibility tests, it shows good platelet adhesion and no significant cytotoxicity to osteoblasts. The limit solid solubility of Zr in Mg is very small (3.8 wt%), which cannot form compounds with Mg, and its strengthening effect is also limited. Zr mainly occurs peritectic reaction in Mg alloys: L → α-Mg + β(Zr). Zr can be added together with Zn, Ag, RE, Th and other elements in Mg–RE alloys that do not contain Al and Mn, as a grain refiner to improve the toughness and corrosion resistance.^202^ Li *et al.* prepared Mg–(0–5)Zr–(0–5)Sr binary and ternary alloys. The alloy has a compressive strength (200–290 MPa) matching natural cortical bone and good biocompatibility. Excessive Zr will introduce unalloyed Mg_17_Sr_2_ phase, which may lead to reduced corrosion resistance and biocompatibility, so Zr content was suggested to be controlled within 5 wt%. The biocompatibility advantage of Zr can be more obvious by adding with Sr at the same time. Therefore, Mg–*x*Zr–*y*Sr and its modifications are more concerned in Zr-containing Mg alloys.^203^ Kiani *et al.* prepared as-extruded Mg–Zr–Sr alloy and found that Sr had a greater effect on grain refinement than Zr. Zr particles are not uniformly distributed in the Mg matrix and the Zr/Mg matrix interface is highly sensitive to corrosion.^204^ The effect of Zr on the grain refinement is shown in Fig. 19. Zr refines the grains of pure Mg, but due to its low solubility, some unalloyed Zr black particles can be observed in the microstructure of Mg–5Zr (Fig. 19(b)). On this basis, 2 wt% Sr further refined grains, but continuous addition of Sr to 5 wt% lead to coarsening of the grains.

Although Zr has been widely used in dental implants and bone implants, its safety still needs further discussion, because its biocompatibility depends on the dose and the valence state of Zr ions.

### 4.4 Essential trace elements

(1) Mn: the concentrations of Mn in whole blood and serum are 200 nmol L^−1^ and 20 nmol L^−1^, respectively. Mn can maintain the normal development of bones, and has the effect of promoting sugar, fat metabolism and anti-oxidation. Excessive Mn lead to Mn poisoning and even psychotic symptoms, such as irritability and hallucinations. The medical term is called manganese mania.^205^

The limiting solid solubility of Mn in Mg is 3.4 wt%, and the contribution to solid solution strengthening is also very small. The mechanism by which Mn improves the corrosion resistance is controversial, and there are two main theories: ① Mn and Fe will form intermetallic compounds and precipitate during the smelting process, thereby reducing the content of Fe in Mg alloys; ② in the process of alloy solidification, Mn can surround Fe, thereby reducing the effect of local cathode. Mn can refine grains and remove Fe, so it is often added in Mg–Al alloys, such as AZ series. The addition of Mn to AZ61,^206^ AZ31 (ref. 207) and AZ21 (ref. 208) has all observed the results of grain refinement, tensile strength and fatigue life improvement. It is believed that Mn itself does not improve the corrosion resistance. The role of Mn in AZ series alloys is mainly to convert Fe and other alloying elements that are harmful to the corrosion resistance into harmless intermediate compounds. Studies have observed that adding 0.2 wt% Mn can inhibit the degradation and stress corrosion.^209^ However, high content of Mn is unfavorable, because Mn and Al can form a large amount of second phase AlMn(Fe) phase with very high electrode potential. Due to the galvanic cell effect, the AlMn(Fe) phase can lead to the occurrence of pitting corrosion, and reduce the fatigue properties.^210^

(2) Zn: Zn is the most widely used alloying element after Al. Like the strengthening mechanism of Mn, Zn improves the corrosion resistance by reducing the common impurities Fe, Ni, Cu, *etc.* in magnesium alloys. The limit solid solubility of Zn in Mg alloys is 6.2 wt%, and decreases significantly with the decrease of temperature. The solid solubility can produce both aging strengthening and solid solution strengthening.^211^ Zn has antibacterial and anti-inflammatory properties, but *in vitro* cell experiments show that excessive intake are toxic to cells in contact. Although Mg–Zn implants do not introduce such large doses, the safe intake needs to be carefully evaluated. One study showed that as-extruded Mg–6Zn alloy aged for 72 h was harmless to L-929 cells in a cytotoxicity test.^212^

**Fig. 20:**
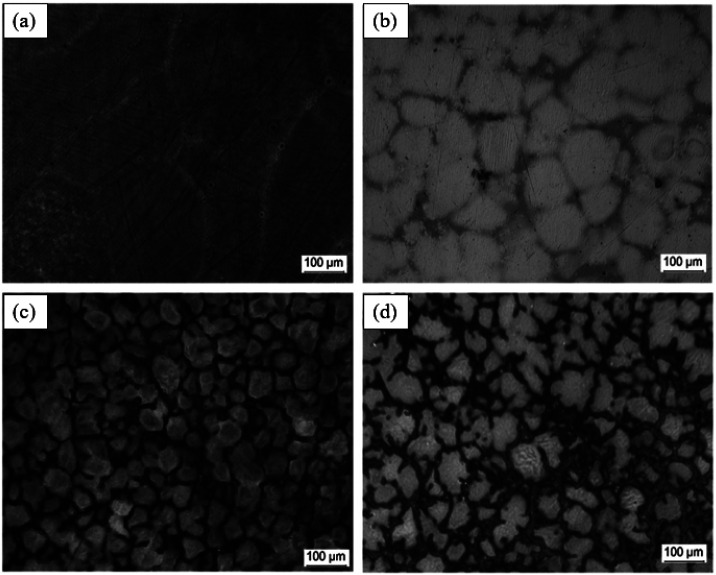
Optical microstructure changes of Mg–Zn alloys (a) pure Mg, (b) Mg–1Zn, (c) Mg–5Zn and (d) Mg–7Zn.^214^

Cai *et al.* cast a series of Mg-*x* wt% Zn (*x* = 1, 5, 7) alloys, the experiments show that the corrosion resistance increases with the increase of Zn (1–5 wt%), and the highest 5 wt% Zn had the best performance. As shown in Fig. 20, the grains were also refined with the addition of Zn. When Zn increased from 5 wt% to 7 wt%, the refining effect became not obvious, and the second phase reticulated MgZn intermediate compounds formed dendrites along the grain boundaries. MgZn intermetallics act as the network structure of the cathode, resulting in accelerated microgalvanic corrosion.^213^ Similar results were obtained in another study using a series of Mg–Zn alloys prepared by powder metallurgy. The tensile strength and elongation of Mg–6Zn meet the implant standards with both good *in vivo* biocompatibility and low *in vitro* degradation rate.^214^

To date, the concentrations of Mn and Zn in degradable magnesium alloys vary greatly due to the difference in the added components, and there is no systematic study of the concentration extremes. Further research is needed on the optimal contents of Mg and Zn in regulating the corrosion resistance, mechanical properties, biocompatibility and biodegradability of biomedical load-bearing Mg alloys.

### 4.5 Harmful elements

Although some alloying elements are classified as “harmful elements”, such as Al, Ce, Nd, *etc.*, excessive intake can lead to mental retardation, convulsions, ataxia, asthma and even death. However, many of them have been successfully used in biological applications, and toxicity tests have shown that these elements cannot be completely excluded from biomedical materials. Therefore, the associated toxicity depends on their content. There are no substances that are absolutely harmful or beneficial to the human body.^215^

(1) Al: as the main alloying element of widely reported AZ series alloys, Al effectively improves the strength through solid solution and precipitation strengthening. The element is partially dissolved in the Mg solid solution, and the rest forms the network Mg_17_Al_12_ or (Mg, Al)_2_Ca phase distributed along the grain boundary, and can also form the granular AlMn(Fe) phase.^216^ Al-added magnesium alloys can form an Al_2_O_3_ film on the surface that is different from Mg(OH)_2_, the film is insoluble in the solution containing Cr to protect the alloy matrix. Another strengthening mechanism is that Al can be partially dissolved in Mg solid solution, and part of it can be precipitated as a continuous network structure or a Mg_17_Al_12_ second phase grown in flakes. AZ91D contains an α-Mg matrix and a β-phase consisting mainly of Mg_17_Al_12_ and eutectic Mg distributed along the dendrite boundaries. These phases have different electrode potentials. When Mg alloy is in contact with corrosive medium, Mg_17_Al_12_ is the positive electrode of the primary battery relative to the Mg matrix, which can accelerate the corrosion. However, due to the inertness of Mg_17_Al_12_ phase, it can resist corrosion in AZ91D.^217^ Observations show that a relatively large amount of reticulated β-phase existing at the grain boundary can effectively hinder the erosion of the corrosive medium. Once the networked β-phase is decomposed, destroyed or not continuously distributed in the Mg matrix during deformation, this protective effect will disappear.^218^ Although Al can effectively refine the structure and improve the corrosion resistance, the harmfulness of excessive intake of Al has become a consensus. The daily intake of adults is about 30–50 mg, and the high Al content will lead to bone calcium deficiency. Al can accumulate in the nervous system, causing neurological disorders and Alzheimer's disease.^219^ Therefore, the use of Al-containing implants should be carefully selected until the long-term effects on the human body are elucidated.

(2) RE: rare earth elements are the collective name of 15 lanthanides, scandium and yttrium, a total of 17 elements. According to the arrangement of the spin directions of the outermost electrons and the size of the ionic radius, rare earth elements can be divided into light rare earth elements (LRE; from La to Eu) and heavy rare earth elements (HRE; from Gd to Lu).^220^

RE elements play a role in solid solution strengthening (impeding the movement of dislocations, the diffusion rate of atoms, strengthening the matrix), fine-grain strengthening (achieving the effect of supercooling the composition, promoting the formation of new nuclei, and inhibiting the growth of α-Mg grains), dispersion strengthening (the formation of fine and dispersed metal compounds during solidification), *etc.*^221^

**Fig. 21:**
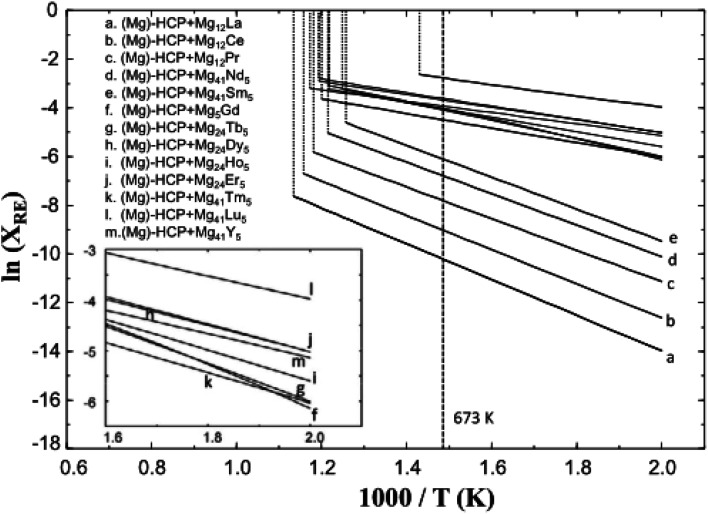
Maximum solubility RE in hcp Mg in the binary Mg–RE alloy systems.^225^

The elements commonly used for alloying include Ce, La, Sn, Y, Nd and Er. Zhang *et al.* studied the effect of Ce/La microalloying on the microstructure changes of solution-treated Mg–Zn–Ca alloys, and found that the addition of Ce/La formed stable CeMg_12_ and Mg_17_La_2_ phases, which effectively inhibited grain growth, and achieved obvious grain refinement effect.^222^ Wang *et al.* added Ce and Sn elements to AZ80. A small amount of Ce improved the hardness, tensile strength and impact toughness of the alloy, and the continuous network coarse β phase becomes fine and uniformly distributed in the grain boundaries. Sn formed a Mg_2_Sn strengthening phase with finer grains, which improves the elongation and tensile strength. The strength, elongation and impact toughness of the alloy decreased with increasing Ce and Sn.^223^ Y and Nd refine grains and improve toughness by changing the deformation (slip and twinning) mechanism, they also bring grain refinement strengthening, dispersion strengthening, solid solution strengthening and precipitation hardening.^224^ The calculated results of the solubility of RE in hcp Mg and Mg–RE intermetallic phases are shown in Fig. 21. The solubility of Y and Nd at 400 °C are 1.95 wt% and 0.11 wt%, respectively. The solubility of LRE is generally lower than that of HRE, and the solubility increases with increasing atomic weight.^225^

The biocompatibility of Mg–RE alloy is the key to determine its large-scale clinical application. Some chronic harmful mechanisms, such as radioactivity of Pm; obvious hepatotoxicity of La, Ce, Pr, Ld; teratogenicity of Nd, Sm, Yb, *etc.*, these REEs bring long-term exposure problems when used as implant materials, limiting the scale of their clinical trials.^226^ ISO 10993-5:2009 stipulates that the retention of more than 70% of cell viability after implantation is considered safe for cells. RE alloys JDBM,^227^ LAE422,^228^ WE43,^229^ ZK21–*x*Sc,^230^*etc.* have all been reported to have 80–100% cell viability on mouse fibroblasts (L-929). However, the only implants based on Mg–RE alloys that have obtained valid clinical test data are the MAGNEZIX series of orthopaedic repair devices (screws, pins and arthrodesis) produced by Syntellix AG, and the Magmaris series (DREAMS) of stents produced by Biotronik.^231^ They have become representatives of successful cases of alloying RE elements.

In summary, RE elements have outstanding advantages and inherent biocompatibility defects in alloying. The toxicity of RE is closely related to its existing state and dose. After being absorbed by the body, the transportation, distribution and excretion of RE depend on its content, compound properties and body's own physiological state. Studies have found that low levels of RE are in an ionic state in the blood, and will be transported to various soft tissues in combination with phosphates, proteins and red blood cell walls. At higher levels of RE, a difficult-to-diffuse colloid or precipitate forms and is excreted in the reticuloendothelial system or in the spleen. The mechanism of excretion is closely related to the element radius, lighter lanthanides are mainly excreted in feces and are therefore processed by the liver, while heavier lanthanides are processed through the urethra (*i.e.* kidneys).^232^ Low levels of RE (La, Gd, Yb, *etc.*) has also been shown to have an activating effect on the sarcoplasmic reticulum Ca^2+^-ATPase, but the increase content of these elements will inhibit enzyme activity.^233^

## 5. Novel coating materials

### 5.1 Graphene-based coating

**Fig. 22:**
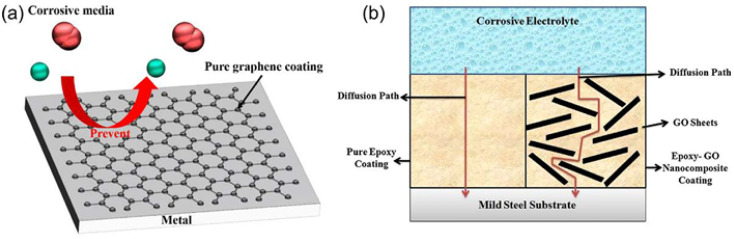
(a) The graphene anti-corrosion coating.^234^ (b) the “labyrinth effect” of GO enhanced epoxy resin coating.^235^

Graphene has a barrier effect on corrosive media such as water and air. Graphene film is known as the thinnest protective coating, with excellent barrier properties and chemical stability, so it has the potential for metal anti-corrosion coating. The corrosion protection effect of graphene materials mainly comes from the following aspects:

(1) Barrier effect: the pure graphene coating covering the surface of the metal substrate plays a role in corrosion protection mainly by the impermeability of graphene, thereby blocking the diffusion of O_2_, water molecules and other corrosive media, as shown in Fig. 22(a). And multilayer graphene will enhance this barrier effect. The corrosion resistance of graphene is mainly affected by the coating and the intrinsic defects of graphene sheets. Corrosive media usually preferentially attack the cracks and defect areas generated during the preparation of the coating, and further diffuse through these areas.^234^

The barrier effect of graphene can also be applied to polymer coatings. The lamellae are dispersed in the polymer matrix, forming complex barriers to prolong the diffusion path of corrosive media, resulting in coatings exhibiting low permeability and slow corrosion rates. Therefore, this phenomenon is called the “maze effect”, as shown in Fig. 22(b). The graphene surface lacks active functional groups, which is easy to agglomerate in the polymer matrix and generate a large number of pores, which weakens the overall protective performance of the coating. While GO or chemically modified RGO showed better dispersion and compatibility with the polymer matrix, resulting in reduced coating porosity.^235,236^

**Fig. 23:**
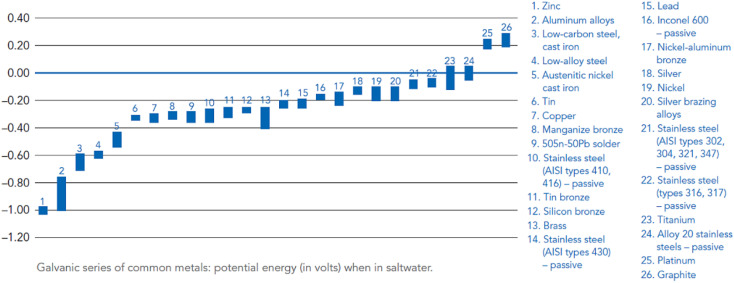
Galvanic series of metals in saltwater noting electrical potential energy.^238^

(2) Enhance the role of sacrificial anode and low standard electrode potential: when graphene is added to a coating that acts as a sacrificial anode, it can act as a cathode phase in the coating and form a conductive network, which can prevent further penetration of the electrolyte. The polymers in the coating can also generate insulating corrosion products, which can reduce the rate of wear of the coating.^237^ Graphite has a lower standard electrode potential than most common metals, making it a cathode material for most metals, as shown in Fig. 23. The high electrical conductivity of graphene is favorable for electron transport in the coating, so corrosion galvanic cells are prone to occur at the graphene–metal interface. The metal is oxidized as the anode, and the electrochemically weak graphene is used as the cathode, and the dissolved oxygen on its surface is reduced. Therefore, the long-term protective effect of graphene-based coatings on metal surfaces is still a hot research topic.^238^

#### 5.1.1 Pure graphene coating

The mainstream graphene film preparation methods include mechanical exfoliation, SiC epitaxial growth, chemical vapor deposition (CVD) and redox processes. Among them, the CVD method has the advantages of simple operation, large product size and high yield. Schriver *et al.* studied the protective effect of single-layer CVD graphene on the Cu surface on the substrate and found that the protective effect of the graphene coating can only last for a short time, and the coating over time accelerates the oxidation and corrosion of the substrate. The reason may be the galvanic corrosion propagation at the machining defects mentioned earlier.^239^ Afterwards, the researchers prepared a multi-layer CVD graphene coating on the surface of Cu for the defects of the single-layer film, and selectively passivated the defects by atomic layer deposition (ALD), and the protection time was effectively elongated. However, since Mg generally have lower melting points than copper, they cannot withstand the high temperatures during deposition, other coating methods should be explored.^240^

#### 5.1.2 Graphene composite anti-corrosion coating

Beyond the single-layer and multilayer pure graphene coatings, graphene is also dispersed into the organic coating matrix as a filler to form a graphene composite anti-corrosion coating. Organic coating is more closely combined with the substrate, and the structure is more complete and denser, while the addition of graphene enhances the barrier property of the coating.^241^ The difficulty in preparing such coatings is to overcome the agglomeration properties of graphene and make it as dispersed as possible. Pure graphene has no functional groups on the surface but a high specific surface area, van der Waals forces between lamellae and π–π interactions, making it easy to agglomerate in aqueous solutions or organics.^242^ To date, there are three main methods to improve the dispersibility of graphene in the coating matrix: physical dispersion, chemical modification and nanoparticle modification of graphene surface, among which chemical modification has achieved better results.

Surface modification of graphene and GO is mainly divided into covalent bonding modification and non-covalent bonding modification.

**Fig. 24:**
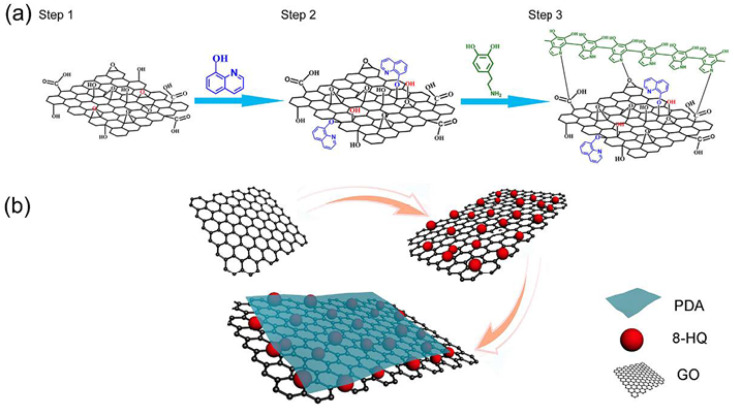
(a) The synthesis process of sandwich-like GO/8-HQ/PDA structures and (b) schematic illustration of the synthesis of the GO/8-HQ/PDA.^244^

Covalent modification is to combine graphene and newly introduced groups in the form of covalent bonds to enhance its performance. The oxygen-containing groups on the surface of GO make it easier to perform covalent functionalization reactions such as epoxy ring opening, carboxyl acylation, diazotization and addition reactions. Palaniappan *et al.* designed octylamine covalently grafted GO materials, and prepared a composite coating of octylamine graphene oxide reinforced epoxy resin on the surface of AZ31B. In the corrosion test, the electrochemical stability of the new coating was improved, and the corrosion rate was alleviated by more than 70%.^243^ Chen *et al.* developed a sandwich structure coating of PDA + 8-HQ + GO, which introduced 8-hydroxyquinoline (8-HQ) on the surface of GO through a ring-opening reaction, followed by dopamine (PDA) through self-reaction. The polymerization was grafted onto the surface of GO/8-HQ to form GO/8-HQ/PDA, as shown in Fig. 24. The coating effectively alleviates the agglomeration of graphene and significantly improves the corrosion resistance of AZ31B in static salt solution.^244^

The non-covalent chemical modification of graphene and GO mainly relies on non-covalent bonds such as π–π interaction force, hydrogen bond, electrostatic force (ionic bond) and van der Waals force. The non-covalent functionalization process is simple and mild, while maintaining the structure and properties of graphene. Zhang *et al.* prepared *in situ* reduced graphene oxide–polyvinyl alcohol composite (GO–PVA) coatings on the surface of magnesium substrates. Hydrogen-bonding interactions were generated between GO and PVA, which were subsequently reduced by low-temperature (120 °C) heat treatment. The prepared coating has a relatively uniform structure, and reduces the corrosion current density of the magnesium matrix to 4% in 3.5 wt% NaCl solution.^245^ However, the non-covalent treatment can change the hybridization of the carbon structure from sp^2^ to sp^3^, which increases the number of defects on the basal plane and affects the mechanical properties of GO nanosheets.

#### 5.1.3 Graphene-based self-assembled coatings

Biomimicry is a method of engineering design by analogy to the characteristics of biological systems. The commonly used biomimetic surface modification of biodegradable Mg alloys is mainly to form an inorganic mineralized calcium phosphate coating close to the inorganic matter of human bone on the surface of the matrix material in the simulated body fluid. The process simulates the mineral apatite in nature. Calcium phosphate is deposited on the surface of Mg substrate. The biomimetic method needs to control a certain temperature and pH value through pretreatment (currently commonly used alkaline washing, acid washing and self-assembly techniques).^246^

**Fig. 25:**
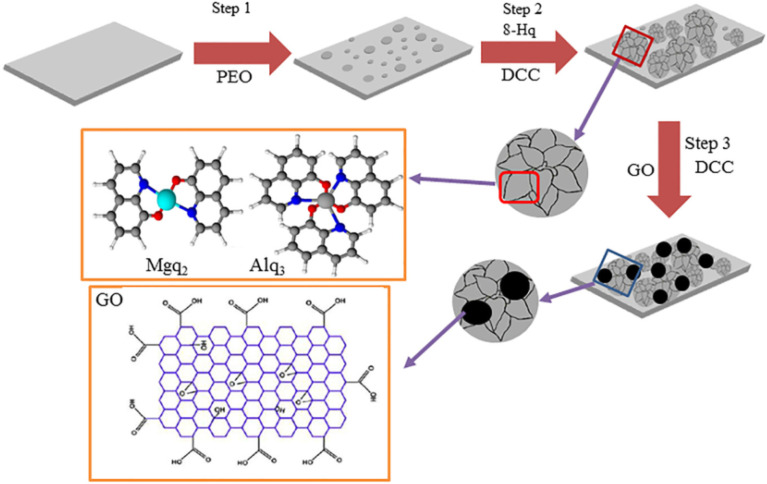
Schematic diagram of the fabrication steps for GO/8-Hq/IC materials.^248^

As a part of biomimetic modification, self-assembly is a technology that enables materials with different charges to form a protective film layer-by-layer on the alloy surface through the interaction of positive and negative electricity. Graphene and GO have a lamellar structure and large specific surface area, which are very suitable for layer-by-layer self-assembly. At the same time, GO exhibits negative charge in aqueous solution, and chemically modified graphene can also exhibit different charge characteristics. Al Zoubi *et al.* developed a self-assembled graphene oxide (GO)/8-hydroxyquinoline (8-Hq)/inorganic coating (IC) hybrid material as a protective material to inhibit the corrosion of the underlying metal (Fig. 25). This organic–inorganic complex has a petal-like structure, and 8-Hq molecules form coordination complexes with metal ions Mg(ii) and Al(iii) in the porous IC coating, which in turn act as junctions for the molecular self-assembly of 8-Hq and GO particles. The physical adsorption between the molecular self-assembly of 8-Hq and GO particles leads to the growth of micron-scale particles with nanoscale features. Compared with bare magnesium, the corrosion rate of the magnesium substrate covered with the composite coating immersed in 3.5% NaCl aqueous solution is greatly slowed down, and the electrochemical stability is significantly enhanced.^247^ Chen *et al.* constructed a phytic acid (PA)–esterified polyethyleneimine (bPEI)–GO multilayer film on the surface of a Mg–1Zn alloy using a hierarchical assembly technique. The self-assembled film has a smooth surface and uniform morphology, which significantly improves the degradation resistance and stress corrosion cracking resistance in Dulbecco's Modified Eagle Medium (DMEM).^248^

#### 5.1.4 Key issues in the application of graphene-based protective coatings

Graphite-based coatings show broad application prospects in metal surface protection, and current research has produced some coatings with excellent corrosion resistance and versatility. But some key questions remain.

(1) Graphene has high chemical inertness and lacks chemical bonding in the coating. Although GO has high chemical activity, its barrier to corrosive media is reduced. Therefore, balancing the reduction degree of graphene and the interfacial bonding of the coating is still a key issue in the construction of corrosion-resistant coatings.^249^

(2) Graphene sheets are very easy to agglomerate in aqueous solutions or organic solvents. Through different structural design or layer modification, the orderly arrangement of graphene or GO sheets can not only reduce coating defects, but also enable the coating to effectively exhibit corrosion resistance. Therefore, dense and ordered graphene-based coatings with high barrier abilities are still the focus of recent research.^250^

(3) Since graphene or GO has a relatively positive electrode potential, when the graphene-based coating is damaged or an electrolyte diffusion channel appears inside, it will cause galvanic corrosion on the metal surface and accelerate the corrosion of the substrate. Therefore, inhibiting galvanic corrosion and prolonging the service life of coatings are still technical challenges.^251^

### 5.2 Polydopamine coating

Traditional alloy surface treatment methods, such as micro-arc oxidation, polymer coating, *etc.*, have poor biocompatibility or poor adhesion to substrates, which hinders the development of related products. In recent years, some methods use high molecular polymers with good bonding properties to combine other functional/bioactive macromolecules to form biomimetic functional layers on the surface of substrate materials through self-assembly. The process is simple, efficient and cost-effective. Inspired by the strong adhesion of marine mussels, polydopamine (PDA) coating has received extensive research attention as a surface modification technology for biomedical materials.^252^

**Fig. 26:**
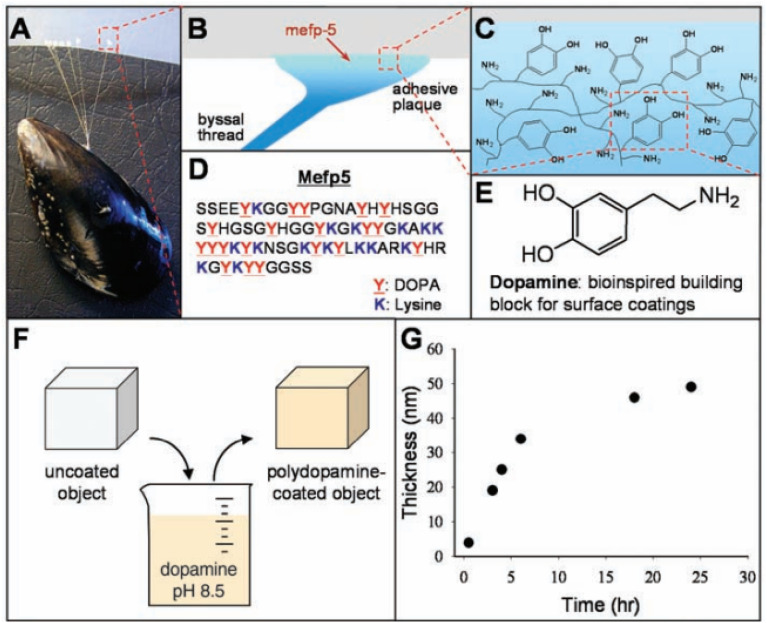
The discovery process of PDA and the structure of DA.

Polydopamine coating is a tightly bound and densely structured polydopamine thin layer. The formation process is as follows: dopamine (DA) contained in the cohesin secreted by marine mussels undergoes self-oxidation, intramolecular/intermolecular rearrangement, cross-linking and other processes in an alkaline aqueous solution, and finally self-polymerizes on the surface of many substrates (precious metals, metal oxides, semiconductors, polymers, ceramics, *etc.*). PDA has the advantages of facile preparation, controllable thickness, strong adhesion to various substrates and degradability. Compared to synthetic polymers such as PCL, PLA and PLGA, DA exhibits enhanced biocompatibility as a natural polymer, mainly due to the absence of highly acidic degradation products. The self-polymerization of DA in aqueous solution was first discovered in 2007 (Fig. 26).^253^

When used as a coating material on the surface of magnesium implants, PDA coating has the following significant advantages.^253^

(1) Enhancement in the corrosion resistance of the substrate. Dopamine self-polymerizes on the substrate material to form a dense polydopamine coating, which forms a cross-linked network structure between molecules; Yu *et al.* reported that the PDA layer has the characteristics of hydrophobicity and negative surface charge in physiological environment. This is theoretically beneficial to reduce water penetration and inhibit Cl^−^ attack on the alloy base, thereby greatly reducing the corrosion rate.^254^

(2) The thickness of the polydopamine coating can be adjusted by controlling the time of the self-polymerization reaction in the solution, and then the adjustment of the different corrosion resistance time of the substrate material can be realized.

(3) Polydopamine grows *in situ* on the surface of the substrate, which is suitable for surface modification of substrate materials with complex surface shapes such as vascular stents.^255^

(4) The PDA layer can effectively enhance the attachment, proliferation and migration of ECs. Pan *et al.* developed a self-assembled PDA coating and used it for AZ31B. The PDA coating effectively reduces the corrosion current by three orders of magnitude. Compared with those grown on unmodified Mg alloys, the value-added curves of ECs were significantly improved, which demonstrated the good compatibility of PDA coatings with ECs.^256^

(5) Strong adhesion and ultra-thin properties (nano-scale). The study shows that the strong adhesion behavior of polydopamine on the sample surface originates from the catechol and amino functional groups of dopamine, and this structure can establish covalent and non-covalent interactions with the organic–inorganic surface, thereby making the polydopamine cross-linked layer to form strong adhesion to material surfaces. This ability to withstand deformation is suitable for the application of materials such as stents that need to withstand large plastic deformation.^257^

Although polydopamine has been extensively studied and applied as a surface modification method, various factors such as preparation conditions restrict the large-scale application of PDA. The PDA coating produced by self-assembly has good properties, but the Mg alloy PDA coating prepared by the traditional method is cumbersome and introduces agglomeration problems.^258^ The commonly used method of preparing PDA coating in Tris–HCl aqueous solution is not suitable for highly chemically active magnesium alloys, and the preparation process will lead to serious corrosion of the substrate. The study by F. Singer *et al.* also showed that the pure PDA coating could not be prepared in the traditional aqueous solution, and the mixture of Mg(OH)_2_/MgO and PDA was obtained.^259^

It should be noted that the blood compatibility of the PDA coating is poor because the imino and quinine groups rapidly adsorb proteins, leading to adverse reactions such as platelet adhesion, aggregation, and coagulation. The researchers mitigated this unfavorable feature by adding heparin.^260^

### 5.3 Bionic superhydrophobic surface

Biomimetic superhydrophobic surfaces have received intense attention from researchers due to numerous potential applications. From the wettability point of view, superhydrophobic surfaces provide an ingenious strategy to solve the corrosion problems. Mg alloys and most of their coatings are hydrophilic, and the contact angles of aqueous solutions at different pH are less than 90°. Therefore, corrosive solutions (such as acids, bases and brine solutions) can easily spread over magnesium or magnesium coatings and corrode the substrate or attack the coating. The spherical water droplets on the superhydrophobic surface can not only prevent the water droplets from spreading or make the water droplets roll away on the macroscopic level, but also form a solid-liquid-gas three-phase contact state at the solid–liquid interface on the microscopic level, which can greatly reduce the contact between substrate, coating and the corrosive liquid.^261^

Early research on superhydrophobic surfaces of Mg alloys mainly focused on the preparation of the surfaces by chemical etching, hydrothermal synthesis, anodization, electrodeposition and micro-arc oxidation. However, the superhydrophobic surfaces prepared by these methods suffer from poor mechanochemical stability. With the development of superhydrophobic coating. The mechanochemical stability of superhydrophobic organic coatings has been significantly improved. Meanwhile, organic superhydrophobic coatings for magnesium alloys have also been developed to further control the degradation process.^262^

#### 5.3.1 Preparation method of superhydrophobic surface

##### 5.3.1.1 Chemical etching

Electroless plating is to replace the metal whose activity sequence is behind Mg to form a micro–nano structure; chemical etching is to prepare the micro–nano structure by acid or chemical oxidation. Corrosive liquid may be involved in the preparation process, and attention should be paid to production protection and waste liquid recovery.

**Fig. 27:**
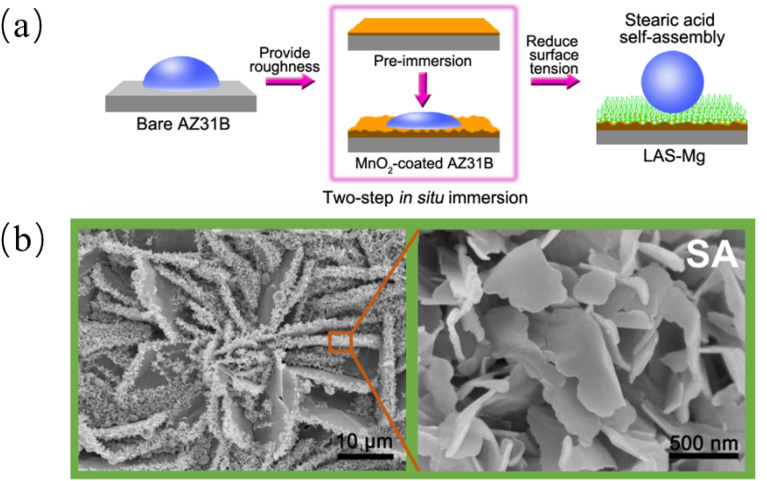
(a) Schematic illustration of the fabrication process of LAS-Mg. (b) SEM images of LAS-Mg.^263^

Xun *et al.* sequentially immersed AZ31B flakes in 0.01 M MnSO_4_, 0.1 M MnSO_4_ and 0.02 M stearic acid in ethanol to prepare a superhydrophobic surface with low adhesion (Fig. 27(a)). As shown in Fig. 27(b), the surface has a large number of randomly and vertically distributed porous wrinkles and stearic acid nanosheets. The superhydrophobic coating reduced the corrosion current of the alloy by two orders of magnitude, indicating that the superhydrophobic surface significantly improves the corrosion resistance of the alloy. WCA was still greater than 150° under UV light and different pH (2–14).^263^

Zang *et al.* used a Mg alloy matrix to react with FeSO_4_ aqueous solution to prepare a rough Fe(OH)_3_ sheet-like structure, and then used stearic acid solution for surface modification to construct a superhydrophobic surface. The measured WCAs on this surface with droplets from pH 1 to 13 were all >160°. The superhydrophobic coating reduces the corrosion current density of alloys by three orders of magnitude.^264^

##### 5.3.1.2 Electrochemical deposition

The process of electrochemical deposition is to first deposit a magnesium alloy in an electrolyte to deposit a micro–nano structure, and then modify it to prepare a superhydrophobic surface. The later improvement is convenient for preparing superhydrophobic surfaces by one-step deposition in electrolytes of low surface energy substances. The advantages of the electrodeposition are simple operation, low cost, high efficiency and large-area fabrication.^265^

**Fig. 28:**
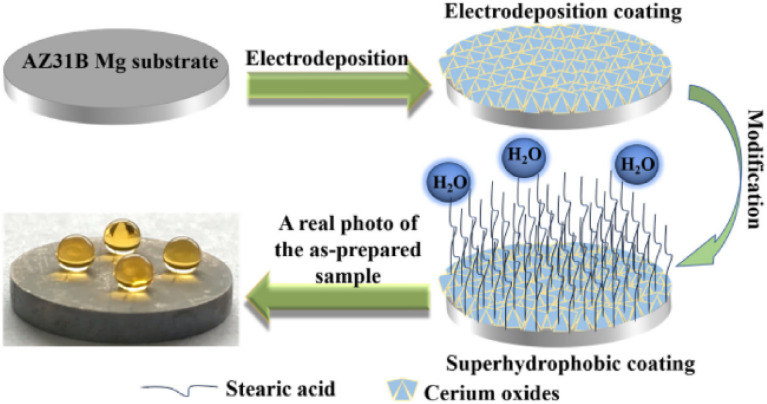
Schematic diagram of preparation process of the superhydrophobic coating formed on the AZ31B Mg substrate.^266^

Liu *et al.* prepared a stearic acid/CeO_2_ bilayer superhydrophobic coating on AZ31B. By using a solution of ethanol : water = 7 : 3 as solvent, the electrolyte containing Ce(NO_3_)_3_·6H_2_O and NH_3_NO_3_, at current densities of 0.65 mA cm^−2^, 1.95 mA cm^−2^ and 3.25 mA cm^−2^, oxidized cerium films were deposited on magnesium alloy samples. Finally, it was modified in 0.05 mol per L stearic acid ethanol solution for 1 h, rinsed with absolute ethanol and dried at room temperature to obtain the coating. The *I*_corr_ of the superhydrophobic coating decreased by two orders of magnitude in the short-term immersion compared with that of the uncoated one, and after immersion in 3.5 wt% NaCl solution for 100 h, the *I*_corr_ of the superhydrophobic coating increased but was still lower than the *I*_corr_ values of uncoated AZ31B in the short-term immersion. The protective performance of the superhydrophobic coating on AZ31B was proved to be over 100 h (Fig. 28).^266^

Liu *et al.* fabricated a superhydrophobic surface by one-step electrodeposition of cerium(iii) nitrate hexahydrate and myristic acid in an ethanol electrolyte solution. The superhydrophobic surface could reduce the *I*_corr_ of magnesium alloys by two orders of magnitude in NaCl, Na_2_SO_4_, NaNO_3_, and NaClO_3_ solutions. The contact angles tested by water droplets with a concentration of 0–5 mol per L NaCl were all greater than 150°. In addition, the WCA tested with water droplets at pH 3 to 13 was in the range of 150° to 157°. The superhydrophobic surface was moved on 1000-grit sandpaper under a pressure of 1.3 kPa. After 400 mm of wear, the WCA of the prepared surface was still higher than 150°. The above results indicate that the superhydrophobic coating not only improves the corrosion performance but also the mechanical properties.^267^

##### 5.3.1.3 Micro-arc oxidation

Micro-arc oxidation (MAO) is a technology in which an alloy is placed in a specific electrolyte and a ceramic film layer is grown *in situ* through micro-plasma discharge. MAO is widely used as a surface treatment due to its low cost, high adhesion of the coating to the substrate and good corrosion and wear resistance.

Zhang *et al.* constructed the surface microstructure through micro-arc oxidation, and finally modified the micro-arc oxidation samples with stearic acid at 99 °C for 0.5 h, 1 h, 3 h and 7 h, and prepared samples with contact angles of 139.5° 142.5°, 144.5° and 155.5° samples. Among them, the sample modified for 7 hours has superhydrophobicity and the best corrosion resistance, and the *I*_corr_ is reduced by three orders of magnitude.^268^

##### 5.3.1.4 Organic coating

Organic superhydrophobic coating has good mechanochemical stability, repairability and excellent anti-corrosion ability. It breaks the limitation of substrate and can prepare superhydrophobic surfaces on different materials under the same preparation process. The organic coating of Mg alloy is relatively specific, and it is often not universal among different alloys, which makes the development more difficult.^269^

Li *et al.* proposed a simple and low-cost method to prepare fluorine-free, mechanochemically durable superhydrophobic coatings by one-step spray coating. The coating composition is epoxy resin, low surface energy material polydimethylsiloxane and modified SiO_2_. The WCA of the coating was 159.5° and the SA was 3.8°. The superhydrophobic coating exhibits excellent mechanochemical durability, high and low temperature stability, and self-healing properties. The *I*_corr_ of the superhydrophobic coating is about 2 orders of magnitude lower than that of the magnesium alloy. After immersion in 3.5 wt% NaCl solution for 336 h, the diameter and impedance modulus of the coated capacitor ring still larger than the original magnesium alloy. Therefore, the coating can provide long-term corrosion protection.^270^

**Fig. 29:**
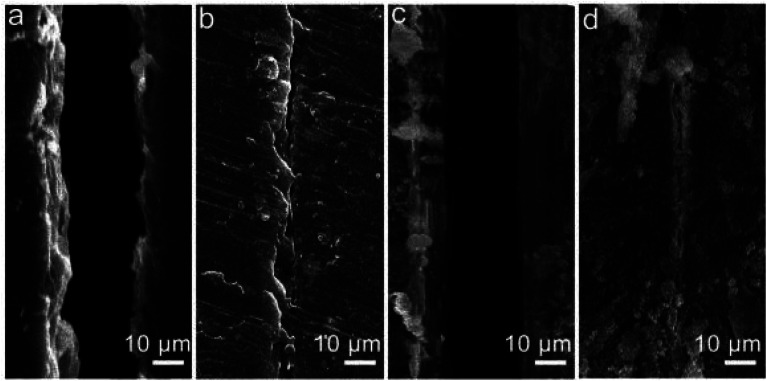
SEM images of the (a) scratched SHEP coating, (b) healed SHEP coating, (c) healed PF-POS@silica coating, and (d) healed SHEP/PF-POS@silica coating.^271^

A self-healing superhydrophobic coating was prepared by Zhao *et al.* The superhydrophobic coating consists of a dense self-healing epoxy resin (SHEP) primer and a superamphiphobic coating topcoat of perfluorodecyl polysiloxane (FD-POS) modified silica (PF-POS@silica). The coatings exhibited good self-healing ability, as shown in Fig. 29, after low-temperature heat treatment, the scratches on SHEP and SHEP/PF-POS@silica coatings could be repaired to a great extent to regain resistance to corrosion. This is because the SHEP layer can effectively drive the repair of the superhydrophobic microstructure, thereby restoring the superhydrophobic properties. This mechanism can effectively reduce the contact area and contact time between the coating and water droplets, thereby effectively preventing the diffusion of corrosive substances such as water, chloride ions and oxygen.^271^

#### 5.3.2 Porous surface

**Fig. 30:**
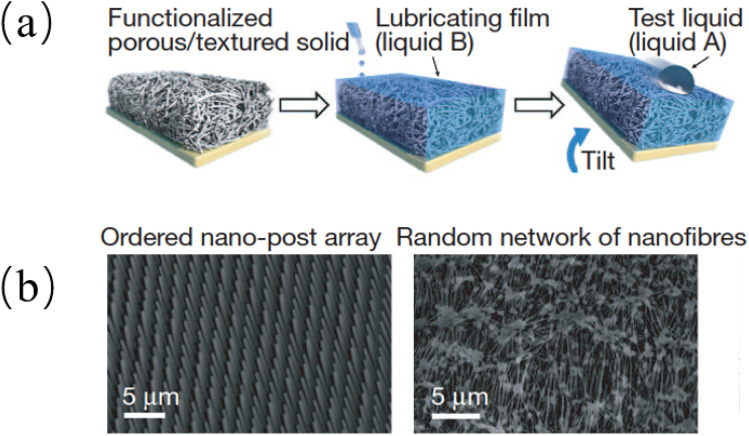
(a) Schematic diagram of SLIPS assembly process; (b) SEM: an epoxy-resin-based nanofabricated post array (left) and a Teflon-based porous nanofibre network (right).^272^

From Fig. 30, in 2011, Wong *et al.*, inspired by the unique wetting properties of the pitcher edge of Nepenthes, used micro/nanostructured storage lubricants to prepare excellently smooth liquid-injected porous surfaces (SLIPS). The SLIPS combines self-healing, hydrophobicity, pressure stability and many other properties. SLIPS is considered a promising multifunctional surface technology with potential applications in liquid repellency, antibacterial, anti-icing and antifouling. It is also of great interest in the field of corrosion protection. Unlike superhydrophobic surfaces, SLIPS forms a solid–oil–water composite interface, and the oil layer greatly reduces the direct contact of water with the solid surface and repels erosive liquids, acting as a corrosion barrier. And because the oil is fluid, it can achieve self-healing properties.^272^ However, in the atmospheric environment, the lubricating oil is easy to evaporate, resulting in a decrease in the non-wetting ability of the surface. This is also one of the factors that limit the widespread application of SLIPS.

Jiang *et al.* designed a novel anti-corrosion system, including plasma electrolytic oxidation (PEO) film, layered double hydroxide (LDH) film, and SLIPS-based magnesium alloy protection system. The PEO film grown *in situ* on the surface of magnesium alloy had moderate corrosion resistance, and the interlayer LDH film loaded with corrosion inhibitors molybdate and SLIPS could seal the PEO film defects. SLIPS provided superior barrier capabilities while improving coating corrosion resistance by refilling damaged areas with lubricating oil. Cl^−^ triggered the release of molybdate in the LDH film for further corrosion inhibition. The system could withstand immersion in 3.5 wt% NaCl solution for 20 days.^273^

Zhang *et al.* prepared a coating with a porous top layer and a dense bottom layer on the surface of AZ31 by a hydrothermal synthesis method. Superhydrophobic surfaces and SLIPS were prepared by modifying the top porous structure and injecting lubricating oil into the porous structure, respectively. The *I*_corr_ of the superhydrophobic surface and SLIPS is 4 and 6 orders of magnitude lower than that of the magnesium alloy, respectively. After prolonged immersion in 3.5 wt% NaCl solution, the superhydrophobic coating can inhibit corrosion for 3 days, and SLIPS can inhibit corrosion for at least 15 days. SLIPS have begun to exhibit significantly better anticorrosion properties than superhydrophobic surfaces.^274^

#### 5.3.3 Development trend of magnesium alloy superhydrophobic coatings

In recent years, the focus of superhydrophobic coatings is on overcoming the durability problems of superhydrophobic coatings, preparing superhydrophobic multifunctional materials, and preparing smart surfaces with tunable wettability, *etc.*

Zang *et al.* reported the preparation of a lotus-like protective superhydrophobic coating on AZ91D. The coating exhibits excellent superhydrophobicity, which can effectively prevent the penetration damage of the coating by water and corrosive ions. Between two extreme wetting behaviors (superhydrophilic and superhydrophobic) was achieved by removing the hydrophobic material (*n*-dodecanethiol) at high temperature (350 °C) and modifying it at room temperature convert. This process can be performed for 18 cycles. The corrosion resistance can be well controlled by the wettability switch, and the corrosion resistance of superhydrophobic surfaces is significantly better than that of superhydrophilic surfaces.^275^ This kind of surface with intelligent regulation of wettability through external stimuli has become one of the important development directions of biomimetic surfaces. During the wettability conversion process, maintaining the long-term corrosion resistance of the coating is crucial for the service of magnesium alloys. Therefore, there is a wide demand for biomimetic surfaces with excellent corrosion resistance and controllable wettability.

From an environmental point of view, superhydrophobic surfaces have made some progress in overcoming traditional problems such as mechanochemical stability and potential environmental pollution. The design of superhydrophobic surfaces that take into account environmental protection, mechanochemical durability, and long-term corrosion resistance can further promote the development of magnesium alloys.

## 6. Challenges and future development

Magnesium-based implants have broad clinical application prospects and great market potential in the fields of bone repair and cardiovascular stents. To overcome the relatively fast degradation rate and inhomogeneity of alloy structures after implantation, there are still many problems with different modification methods. Elements that negatively affect the mechanical properties and corrosion rate of the alloy should be discarded under the action of complex alloying mechanism, and the long-term toxicity of human trace elements (such as Li, Al, Sc, RE elements, *etc.*) must be evaluated. The contribution of biomimetic materials and graphene to the corrosion resistance of alloys has not yet been fully explored, while their biosafety has also to be evaluated.

The development trend of medical magnesium alloys in the future may go through the process of alloying → microalloying/plain → high purification in terms of composition.

After several years of research on alloying, breakthroughs and attempts have been made in the types and quantities of alloys added, more complex microstructures have been obtained, and the types and volume fractions of the second phases formed have also increased. Alloying often has a significant effect on improving mechanical properties, but also brings the side effect of corrosion, especially pitting susceptibility. The pros and cons of over-alloying are now evident. Therefore, the process of microalloying is to explore the interference on the original performance after appropriately reducing the element content. Plain is to manufacture sustainable materials by regulating the defects of different scales of materials without changing the composition. Both methods are significant for material cost control and sustainable development.

High purification means that impurity elements such as Fe, Co, Ni, Cu, *etc.* are controlled in the amount that interferes with the performance of the alloy with the least amount. The purpose can be achieved by the following means: (1) the peritectic reaction is used to replace the eutectic reaction as much as possible. The peritectic reaction of Mg is promoted by adding certain alloying elements such as Mn, Zr, Ti, *etc.* in an amount not exceeding the solid solution limit. (2) When eutectic elements must be selected for alloying, the higher the solid solubility of the metals of the other components, the better. The compound adjacent to the solid solution phase region is better with higher stability, and it is suggested to control the eutectic point to be far away from Mg in the phase diagram. (3) Adding specific alloying elements to remove impurities, such as adding Mn to remove Fe.

In terms of function, the development trend of medical Mg alloys is to change from a purely structural consideration to an integrated design of structure and function. For example, the DREAMS series stents have added a drug-loading layer to reduce intimal hyperplasia.

In the research of medical Mg alloy coating materials, the issues that need to be paid more attention in the future are: (1) the surface coating material should have both biocompatibility and long-term protection against corrosion, and can inhibit the excessive release of hydrogen during the degradation of magnesium alloys. (2) According to the specific requirements of different implanted parts of the human body, develop specific modified coatings and preparation technologies. For example, the environments of human bone and blood are very different. Generally, the requirements for material properties in the blood environment are significantly higher than those in the bone environment. Both animal and human experiments have shown that the corrosion rate of magnesium alloys in blood is significantly higher than that in the bone environment, so it is necessary to develop different types of coating materials. (3) The coating materials should have controlled biodegradability. In-depth and meticulous research and scientific evaluation must be carried out for the metabolism and absorption pathways of coating material degradation products in the human body and their effects on major internal organs.

## Conflicts of interest

There are no conflicts to declare.

## Supplementary Material

## References

[cit1] Witte F. (2010). Acta Biomater..

[cit2] Virtanen S. (2011). Mater. Sci. Eng., B.

[cit3] Guan R.-g., Cipriano A. F., Zhao Z.-y., Lock J., Tie D., Zhao T., Cui T., Liu H. (2013). Mater. Sci. Eng., C.

[cit4] Vormann J. (2003). Mol. Aspects Med..

[cit5] Swaminathan R. (2003). Clin. Biochem. Rev..

[cit6] Rho J.-Y., Kuhn-Spearing L., Zioupos P. (1998). Med. Eng. Phys..

[cit7] Meng X., Jiang Z., Zhu S., Guan S. (2020). J. Alloys Compd..

[cit8] Totemeier T. C. (2005). J. Therm. Spray Technol..

[cit9] Kang Y., Wu D., Chen R., Han E. (2014). J. Magnesium Alloys.

[cit10] Munitz A., Cotler C., Stern A., Kohn G. (2001). Mater. Sci. Eng., A.

[cit11] Arrazola P.-J., Garay A., Iriarte L.-M., Armendia M., Marya S., Le Maître F. (2009). J. Mater. Process. Technol..

[cit12] Persaud-Sharma D., McGoron A. (2011). J. Biomimetics, Biomater., Tissue Eng..

[cit13] Lee S.-H., Takahashi E., Nomura N., Chiba A. (2005). Mater. Trans..

[cit14] Zhao Z., Chen Q., Tang Z., Hu C. (2010). J. Alloys Compd..

[cit15] Athanasiou K. A., Agrawal C. M., Barber F. A., Burkhart S. S. (1998). Arthroscopy.

[cit16] Zhao D., Wang T., Nahan K., Guo X., Zhang Z., Dong Z., Chen S., Chou D.-T., Hong D., Kumta P. N. (2017). Acta Biomater..

[cit17] Zhang Y., Hinton B., Wallace G., Liu X., Forsyth M. (2012). Corros. Eng., Sci. Technol..

[cit18] Heiden M., Walker E., Stanciu L. (2015). J. Biotechnol. Biomater..

[cit19] Pogorielov M., Husak E., Solodivnik A., Zhdanov S. (2017). Interventional Medicine and Applied Science.

[cit20] Zheng Y. F., Gu X. N., Witte F. (2014). Mater. Sci. Eng., R.

[cit21] Cao F., Shi Z., Hofstetter J., Uggowitzer P. J., Song G., Liu M., Atrens A. (2013). Corros. Sci..

[cit22] Jafari S., Raman R. K. S., Davies C. H. J., Hofstetter J., Uggowitzer P. J., Loffler J. F. (2017). J. Mech. Behav. Biomed. Mater..

[cit23] Xie J., Zhang J., Zhang Z., Qiu X., Zhang H., Zhang H., Jiao X., Wu X., Wu R. (2022). J. Mater. Res. Technol..

[cit24] Bahmani A., Arthanari S., Shin K. S. (2019). Int. J. Adv. Manuf. Technol..

[cit25] Wang X., Chen Z., Guo E., Liu X., Kang H., Wang T. (2021). J. Alloys Compd..

[cit26] Wang X., Chen Z., Ren J., Kang H., Guo E., Li J., Wang T. (2020). Corros. Sci..

[cit27] El-Mahallawy N., Palkowski H., Klingner A., Diaa A., Shoeib M. (2020). Mater. Today Commun..

[cit28] Li J. A., Chen L., Zhang X. Q., Guan S. K. (2020). Mater. Sci. Eng., C.

[cit29] Su C., Wang J., Hu H., Wen Y., Liu S., Ma K. (2021). J. Alloys Compd..

[cit30] Zengin H., Turen Y., Ahlatci H., Sun Y. (2020). Rare Met..

[cit31] Jafari H., Heidari E., Barabi A., Dashti Kheirabadi M. (2018). Acta Metall. Sin. (Engl. Lett.).

[cit32] Dai J., Dong Q., Nie Y., Jia Y., Chu C., Zhang X. (2022). Mater. Des..

[cit33] Liu B.-s., Wang H.-H., Zhang Y.-z., Yang Y.-x., Ren X.-x., Du H.-y., Hou L.-f., Wei Y.-h., Song G.-l. (2021). J. Phys. Chem. Solids.

[cit34] Yao H., Li Y., Wee A. (2000). Appl. Surf. Sci..

[cit35] Liu M., Zanna S., Ardelean H., Frateur I., Schmutz P., Song G., Atrens A., Marcus P. (2009). Corros. Sci..

[cit36] Nordlien J. H., Ono S., Masuko N., Nisancioglu K. (1997). Corros. Sci..

[cit37] Czerwinski F. (2012). JOM.

[cit38] Esmaily M., Svensson J., Fajardo S., Birbilis N., Frankel G., Virtanen S., Arrabal R., Thomas S., Johansson L. (2017). Prog. Mater. Sci..

[cit39] Zong Y., Yuan G., Zhang X., Mao L., Niu J., Ding W. (2012). Mater. Sci. Eng., B.

[cit40] Xin Y., Hu T., Chu P. (2011). Acta Biomater..

[cit41] Liu X., Chu P. K., Ding C. (2010). Mater. Sci. Eng., R.

[cit42] Wei J., Li B., Jing L., Tian N., Zhao X., Zhang J. (2020). Chem. Eng. J..

[cit43] Heise S., Virtanen S., Boccaccini A. R. (2016). J. Biomed. Mater. Res., Part A.

[cit44] Yang L., Xiao X., Shen S., Lama J., Hu M., Jia F., Han Z., Qu H., Huang L., Wang Y. (2022). ACS Appl. Nano Mater..

[cit45] Xu C. L., Song F., Wang X.-L., Wang Y.-Z. (2017). Chem. Eng. J..

[cit46] Jia S., Lu X., Luo S., Qing Y., Yan N., Wu Y. (2018). Chem. Eng. J..

[cit47] Cao M., Jin X., Peng Y., Yu C., Li K., Liu K., Jiang L. (2017). Adv. Mater..

[cit48] Zhi J.-H., Zhang L.-Z., Yan Y., Zhu J. (2017). Appl. Surf. Sci..

[cit49] Zhang Y., Feyerabend F., Tang S., Hu J., Lu X., Blawert C., Lin T. (2017). Mater. Sci. Eng., C.

[cit50] Della Vecchia N. F., Avolio R., Alfè M., Errico M. E., Napolitano A., d'Ischia M. (2013). Adv. Funct. Mater..

[cit51] Liu Y., Zheng Y., Chen X. H., Yang J. A., Pan H., Chen D., Wang L., Zhang J., Zhu D., Wu S. (2019). Adv. Funct. Mater..

[cit52] Ding Y., Wen C., Hodgson P., Li Y. (2014). J. Mater. Chem. B.

[cit53] Zhao X., Shi L.-l., Xu J. (2013). Mater. Sci. Eng., C.

[cit54] Gu X., Xie X., Li N., Zheng Y., Qin L. (2012). Acta Biomater..

[cit55] Zhao C., Pan F., Zhang L., Pan H., Song K., Tang A. (2017). Mater. Sci. Eng., C.

[cit56] Li M., Yang X., Wang W., Zhang Y., Wan P., Yang K., Han Y. (2017). Mater. Sci. Eng., C.

[cit57] Dong J., Tan L., Yang J., Wang Y., Chen J., Wang W., Zhao D., Yang K. (2018). Mater. Technol..

[cit58] Minárik P., Čížek J., Veselý J., Hruška P., Hadzima B., Král R. (2017). Mater. Charact..

[cit59] Minárik P., Jablonská E., Král R., Lipov J., Ruml T., Blawert C., Hadzima B., Chmelík F. (2017). Mater. Sci. Eng., C.

[cit60] Kleer N., Julmi S., Gartzke A. K., Augustin J., Feichtner F., Waselau A. C., Klose C., Maier H. J., Wriggers P., Meyer-Lindenberg A. (2019). Materialia.

[cit61] Xu G., Zhang L., Liu L., Du Y., Zhang F., Xu K., Liu S., Tan M., Jin Z. (2016). J. Magnesium Alloys.

[cit62] Pereira G. S., Koga G. Y., Avila J. A., Bittencourt I. M., Fernandez F., Miyazaki M. H., Botta W. J., Bose Filho W. W. (2021). Mater. Chem. Phys..

[cit63] Peeters P., Bosiers M., Verbist J., Deloose K., Heublein B. (2005). J. Endovasc. Ther..

[cit64] Haude M., Erbel R., Erne P., Verheye S., Degen H., Böse D., Vermeersch P., Wijnbergen I., Weissman N., Prati F. (2013). Lancet.

[cit65] Haude M., Ince H., Abizaid A., Toelg R., Lemos P. A., von Birgelen C., Christiansen E. H., Wijns W., Neumann F.-J., Kaiser C. (2016). Eur. Heart J..

[cit66] Kitabata H., Waksman R., Warnack B. (2014). Cardiovasc. Revascularization Med..

[cit67] Marco I., Feyerabend F., Willumeit-Römer R., Van der Biest O. (2016). Mater. Sci. Eng., C.

[cit68] Johnson I., Jiang W., Liu H. (2017). Sci. Rep..

[cit69] Liu C., Wang Y., Zeng R., Zhang X., Huang W., Chu P. (2010). Corros. Sci..

[cit70] Witte F., Mantovani D. (2013). Acta Biomater..

[cit71] Scheideler L., Füger C., Schille C., Rupp F., Wendel H.-P., Hort N., Reichel H., Geis-Gerstorfer J. (2013). Acta Biomater..

[cit72] Wang J., Witte F., Xi T., Zheng Y., Yang K., Yang Y., Zhao D., Meng J., Li Y., Li W. (2015). Acta Biomater..

[cit73] Oberhauser J. P., Hossainy S., Rapoza R. J. (2009). EuroIntervention.

[cit74] Waksman R., Erbel R., Di Mario C., Bartunek J., de Bruyne B., Eberli F. R., Erne P., Haude M., Horrigan M., Ilsley C. (2009). JACC: Cardiovasc. Interventions.

[cit75] Wittchow E., Adden N., Riedmüller J., Savard C., Waksman R., Braune M. (2013). EuroIntervention.

[cit76] Zhang Y., Bourantas C. V., Farooq V., Muramatsu T., Diletti R., Onuma Y., Garcia-Garcia H. M., Serruys P. W. (2013). Medical Devices.

[cit77] Wu S., Liu X., Yeung K. W., Liu C., Yang X. (2014). Mater. Sci. Eng., R.

[cit78] Weng W., Biesiekierski A., Li Y., Dargusch M., Wen C. (2021). Acta Biomater..

[cit79] Seitz J.-M., Lucas A., Kirschner M. (2016). JOM.

[cit80] Waizy H., Diekmann J., Weizbauer A., Reifenrath J., Bartsch I., Neubert V., Schavan R., Windhagen H. (2014). J. Biomater. Appl..

[cit81] Biber R. , Pauser J., Brem M., Bail H. J., Trauma Case Reports, 2017, vol. 8, pp. 11–15 10.1016/j.tcr.2017.01.012 PMC5883197 29644307

[cit82] Peiwei G., Xiaolin L., Shaochun J., Hui Z., Chunxing G. (2008). Mater. Lett..

[cit83] Hakimi O., Ventura Y., Goldman J., Vago R., Aghion E. (2016). Mater. Sci. Eng., C.

[cit84] Vickers N. J. (2017). Curr. Biol..

[cit85] Aghion E., Levy G. (2010). J. Mater. Sci..

[cit86] Mohammadi J., Ghoreishi M., Behnamian Y. (2014). Mater. Res..

[cit87] Zhang H., Hu S., Wang Z., Liang Y. (2015). Mater. Des..

[cit88] Zhang X., Yuan G., Mao L., Niu J., Ding W. (2012). Mater. Lett..

[cit89] Zhang J., Hiromoto S., Yamazaki T., Niu J., Huang H., Jia G., Li H., Ding W., Yuan G. (2016). J. Biomed. Mater. Res., Part A.

[cit90] Zhang J., Li H., Wang W., Huang H., Pei J., Qu H., Yuan G., Li Y. (2018). Acta Biomater..

[cit91] Xie K., Wang L., Guo Y., Zhao S., Yang Y., Dong D., Ding W., Dai K., Gong W., Yuan G. (2021). J. Orthop. Transl..

[cit92] Liu M., Schmutz P., Uggowitzer P. J., Song G., Atrens A. (2010). Corros. Sci..

[cit93] Hänzi A. C., Gerber I., Schinhammer M., Löffler J. F., Uggowitzer P. J. (2010). Acta Biomater..

[cit94] Wang L., Jiang J., Liu H., Saleh B., Ma A. (2020). J. Magnesium Alloys.

[cit95] Chou D.-T., Hong D., Saha P., Ferrero J., Lee B., Tan Z., Dong Z., Kumta P. N. (2013). Acta Biomater..

[cit96] Kubásek J., Dvorský D., Čavojský M., Vojtěch D., Beronská N., Fousová M. (2017). J. Mater. Sci. Technol..

[cit97] Ogawa Y., Ando D., Sutou Y., Koike J. (2016). Science.

[cit98] MacBarb R. F., Makris E. A., Hu J. C., Athanasiou K. A. (2013). Acta Biomater..

[cit99] Aboutalebianaraki N., Neal C. J., Seal S., Razavi M. (2022). J. Funct. Biomater..

[cit100] Li L.-Y., Cui L.-Y., Zeng R.-C., Li S.-Q., Chen X.-B., Zheng Y., Kannan M. B. (2018). Acta Biomater..

[cit101] Virtanen S. (2011). Mater. Sci. Eng., B.

[cit102] Guo K. W. (2011). Recent Pat. Corros. Sci..

[cit103] Walter R., Kannan M. B. (2011). Mater. Des..

[cit104] Li L.-Y., Liu B., Zeng R.-C., Li S.-Q., Zhang F., Zou Y.-H., Jiang H. G., Chen X.-B., Guan S.-K., Liu Q.-Y. (2018). Front. Mater. Sci..

[cit105] Martin H. J., Horstemeyer M., Wang P. T. (2010). Corros. Sci..

[cit106] van Gaalen K., Gremse F., Benn F., McHugh P. E., Kopp A., Vaughan T. J. (2022). Bioact. Mater..

[cit107] Merson D., Vasilev E., Markushev M., Vinogradov A. (2017). Letters on Materials.

[cit108] Wang P., Liu J., Shen S., Li Q., Luo X., Xiong P., Gao S., Yan J., Cheng Y., Xi T. (2019). ACS Biomater. Sci. Eng..

[cit109] Kirkland N., Lespagnol J., Birbilis N., Staiger M. (2010). Corros. Sci..

[cit110] Song Y., Shan D., Han E.-H. (2017). J. Mater. Sci. Technol..

[cit111] Song Y., Shan D., Chen R., Han E.-H. (2009). Corros. Sci..

[cit112] Yang W., Liu Z., Huang H. (2021). Corros. Sci..

[cit113] Yang L., Huang Y., Feyerabend F., Willumeit R., Mendis C., Kainer K., Hort N. (2013). Acta Biomater..

[cit114] Ma C., Peng G., Nie L., Liu H., Guan Y. (2018). Appl. Surf. Sci..

[cit115] Wang L., Jiang J., Liu H., Saleh B., Ma A. (2020). J. Magnesium Alloys.

[cit116] Liu Y., Liu X., Zhang Z., Farrell N., Chen D., Zheng Y. (2019). Corros. Sci..

[cit117] Song G., Johannesson B., Hapugoda S., StJohn D. (2004). Corros. Sci..

[cit118] Zeng R., Kainer K. U., Blawert C., Dietzel W. (2011). J. Alloys Compd..

[cit119] Rongchang Z., Enhou H., Wei K. (2007). J. Mater. Sci. Technol..

[cit120] Chen Z., Li H., Liang X., Zhao M.-C., Zhang K., Atrens A. (2022). J. Magnesium Alloys.

[cit121] Song Y., Shan D., Chen R., Han E.-H. (2010). Corros. Sci..

[cit122] Wang S. D., Xu D., Chen X., Han E., Dong C. (2015). Corros. Sci..

[cit123] Gu X., Zhou W., Zheng Y., Cheng Y., Wei S., Zhong S., Xi T., Chen L. (2010). Acta Biomater..

[cit124] Shiozawa K., Kashiwagi T., Murai T., Takahashi T. (2010). Procedia Eng..

[cit125] Liu Y., Liu Z., Liu L., Xue H., Wang Q., Zhang D. (2021). Adv. Eng. Mater..

[cit126] Jafari S., Raman R. K. S., Davies C. H. J., Hofstetter J., Uggowitzer P. J., Loffler J. F. (2017). J. Mech. Behav. Biomed. Mater..

[cit127] Catar R., Altun H. (2019). Open Chem..

[cit128] Jafari S., Raman R. S., Davies C. H. (2018). Eng. Fract. Mech..

[cit129] Deshpande K. B. (2011). Electrochim. Acta.

[cit130] Yan D., Wang Y., Liu J., Song D., Zhang T., Liu J., He F., Zhang M., Wang J. (2020). J. Alloys Compd..

[cit131] Abdalla M., Joplin A., Elahinia M., Ibrahim H. (2020). Corrosion and Materials Degradation.

[cit132] Deng M., Höche D., Lamaka S. V., Wang L., Zheludkevich M. L. (2019). Corros. Sci..

[cit133] Kimura S. and Yasuda N., Materials Science Forum, Trans Tech Publ, 2018, pp. 1123–1126

[cit134] Sun M., Yerokhin A., Bychkova M. Y., Shtansky D., Levashov E., Matthews A. (2016). Corros. Sci..

[cit135] Williams G., ap Llwyd Dafydd H., Grace R. (2013). Electrochim. Acta.

[cit136] Lamaka S., Karavai O., Bastos A., Zheludkevich M., Ferreira M. (2008). Electrochem. Commun..

[cit137] Williams G., McMurray H. N. (2008). J. Electrochem. Soc..

[cit138] Patel V., Li W., Andersson J., Li N. (2022). J. Mater. Res. Technol..

[cit139] Wang Y., Cheng G., Wu W., Qiao Q., Li Y., Li X. (2015). Appl. Surf. Sci..

[cit140] Zai W., Su Y., Man H. C., Lian J., Li G. (2019). Appl. Surf. Sci..

[cit141] Wang H., Song Y., Yu J., Shan D., Han H. (2017). J. Electrochem. Soc..

[cit142] Williams G., Grace R. (2011). Electrochim. Acta.

[cit143] Kannan M. B., Dietzel W., Blawert C., Atrens A., Lyon P. (2008). Mater. Sci. Eng., A.

[cit144] He X., Yan Z., Liang H., Wei Y. (2017). J. Mater. Eng. Perform..

[cit145] Raman R. S., Harandi S. E. (2017). Materials.

[cit146] Liao H.-j., Zhou X.-f., Li H.-z., Min D., Liang X.-p., Liu R.-m. (2015). Trans. Nonferrous Met. Soc. China.

[cit147] Zeng R., Han E., Ke W. (2012). Int. J. Fatigue.

[cit148] Bhuiyan M. S., Mutoh Y., Murai T., Iwakami S. (2010). Eng. Fract. Mech..

[cit149] Gou G., Zhang M., Chen H., Chen J., Li P., Yang Y. (2015). Mater. Des..

[cit150] Marques M. R., Loebenberg R., Almukainzi M. (2011). Dissolution Technol..

[cit151] Bairagi D., Mandal S. (2022). J. Magnesium Alloys.

[cit152] Taltavull C., Shi Z., Torres B., Rams J., Atrens A. (2014). J. Mater. Sci.: Mater. Med..

[cit153] Ye L., Li F., Wu T., Li Y. (2016). Korean J. Chem. Eng..

[cit154] Esmaily M., Svensson J., Fajardo S., Birbilis N., Frankel G., Virtanen S., Arrabal R., Thomas S., Johansson L. (2017). Prog. Mater. Sci..

[cit155] Xin Y., Huo K., Tao H., Tang G., Chu P. K. (2008). Acta Biomater..

[cit156] Liang M.-j., Wu C., Ma Y., Wang J., Dong M., Dong B., Liao H.-h., Fan J., Guo Z. (2021). Mater. Sci. Eng., C.

[cit157] Song Y., Shan D., Han E. (2008). Mater. Lett..

[cit158] Marco I., Myrissa A., Martinelli E., Feyerabend F., Willumeit-Römer R., Weinberg A., Van der Biest O. (2017). Eur. Cells Mater..

[cit159] Chen Y., Xu Z., Smith C., Sankar J. (2014). Acta Biomater..

[cit160] Yang L., Hort N., Willumeit R., Feyerabend F. (2012). Corros. Eng., Sci. Technol..

[cit161] Zhang J., Kong N., Shi Y., Niu J., Mao L., Li H., Xiong M., Yuan G. (2014). Corros. Sci..

[cit162] Harandi S. E., Banerjee P. C., Easton C. D., Raman R. S. (2017). Mater. Sci. Eng., C.

[cit163] Li T., He Y., Zhou J., Tang S., Yang Y., Wang X. (2018). Mater. Lett..

[cit164] Yamamoto A., Hiromoto S. (2009). Mater. Sci. Eng., C.

[cit165] Wang Y., Cui L.-Y., Zeng R.-C., Li S.-Q., Zou Y.-H., Han E.-H. (2017). Materials.

[cit166] Li L.-Y., Liu B., Zeng R.-C., Li S.-Q., Zhang F., Zou Y.-H., Jiang H. G., Chen X.-B., Guan S.-K., Liu Q.-Y. (2018). Front. Mater. Sci..

[cit167] Mei D., Lamaka S. V., Feiler C., Zheludkevich M. L. (2019). Corros. Sci..

[cit168] Cui L.-Y., Li X.-T., Zeng R.-C., Li S.-Q., Han E.-H., Song L. (2017). Front. Mater. Sci..

[cit169] Li L.-Y., Liu B., Zeng R.-C., Li S.-Q., Zhang F., Zou Y.-H., Jiang H. G., Chen X.-B., Guan S.-K., Liu Q.-Y. (2018). Front. Mater. Sci..

[cit170] Heiden M., Walker E., Stanciu L. (2015). J. Biotechnol. Biomater..

[cit171] Liang J., Srinivasan P. B., Blawert C., Dietzel W. (2010). Corros. Sci..

[cit172] Altun H., Sen S. (2004). Mater. Des..

[cit173] Freire L., Catarino M. A., Godinho M., Ferreira M., Ferreira M., Simões A., Montemor M. (2012). Cem. Concr. Compos..

[cit174] Li S., Bacco A. C., Birbilis N., Cong H. (2016). Corros. Sci..

[cit175] Maltseva A., Shkirskiy V., Lefèvre G., Volovitch P. (2019). Corros. Sci..

[cit176] Sun K., Gao H., Hu J., Yan Y. (2021). Corros. Sci..

[cit177] Kirkland N. T. and Birbilis N., Magnesium Biomaterials: Design, Testing, and Best Practice, Springer, 2014

[cit178] Wang J., Giridharan V., Shanov V., Xu Z., Collins B., White L., Jang Y., Sankar J., Huang N., Yun Y. (2014). Acta Biomater..

[cit179] Lévesque J., Hermawan H., Dubé D., Mantovani D. (2008). Acta Biomater..

[cit180] Peron M., Torgersen J., Berto F. (2019). Procedia Struct. Integr..

[cit181] Zhu T., Yu Y., Yang J., Shen Y., He L., Xiong Y. (2021). Mater. Chem. Phys..

[cit182] Jana A., Das M., Balla V. K. (2022). J. Biomed. Mater. Res., Part A.

[cit183] Myrissa A., Agha N. A., Lu Y., Martinelli E., Eichler J., Szakacs G., Kleinhans C., Willumeit-Römer R., Schäfer U., Weinberg A.-M. (2016). Mater. Sci. Eng., C.

[cit184] Xin Y., Hu T., Chu P. (2011). Acta Biomater..

[cit185] Waizy H., Seitz J.-M., Reifenrath J., Weizbauer A., Bach F.-W., Meyer-Lindenberg A., Denkena B., Windhagen H. (2013). J. Mater. Sci..

[cit186] Garg S., Bourantas C., Serruys P. W. (2013). Nat. Rev. Cardiol..

[cit187] Prabhu D. B., Gopalakrishnan P., Ravi K. (2017). Mater. Des..

[cit188] Sanchez A. H. M., Luthringer B. J., Feyerabend F., Willumeit R. (2015). Acta Biomater..

[cit189] Ascencio M., Pekguleryuz M., Omanovic S. (2014). Corros. Sci..

[cit190] Loukil N. , Magnesium Alloys Structure and Properties, 2021

[cit191] Azzeddine H., Hanna A., Dakhouche A., Rabahi L., Scharnagl N., Dopita M., Brisset F., Helbert A.-L., Baudin T. (2020). J. Alloys Compd..

[cit192] Imandoust A., Barrett C. D., Al-Samman T., Tschopp M. A., Essadiqi E., Hort N., El Kadiri H. (2018). Metall. Mater. Trans. A.

[cit193] Sharma S. K., Saxena K. K., Malik V., Mohammed K. A., Prakash C., Buddhi D., Dixit S. (2022). Crystals.

[cit194] Seong J., Kim W. (2015). Acta Biomater..

[cit195] Liu H., Cao F., Song G.-L., Zheng D., Shi Z., Dargusch M. S., Atrens A. (2019). J. Mater. Sci. Technol..

[cit196] Harandi S. E., Mirshahi M., Koleini S., Idris M. H., Jafari H., Kadir M. R. A. (2013). Mater. Res..

[cit197] Zeng R.-C., Qi W.-C., Cui H.-Z., Zhang F., Li S.-Q., Han E.-H. (2015). Corros. Sci..

[cit198] Rad H. R. B., Idris M. H., Kadir M. R. A., Farahany S. (2012). Mater. Des..

[cit199] Brar H. S., Wong J., Manuel M. V. (2012). J. Mech. Behav. Biomed. Mater..

[cit200] Gu X., Xie X., Li N., Zheng Y., Qin L. (2012). Acta Biomater..

[cit201] Bornapour M., Muja N., Shum-Tim D., Cerruti M., Pekguleryuz M. (2013). Acta Biomater..

[cit202] Chen Y., Xu Z., Smith C., Sankar J. (2014). Acta Biomater..

[cit203] Li Y., Wen C., Mushahary D., Sravanthi R., Harishankar N., Pande G., Hodgson P. (2012). Acta Biomater..

[cit204] Kiani F., Lin J., Vahid A., Munir K., Wen C., Li Y. (2022). Mater. Sci. Eng., A.

[cit205] Zabłocka-Słowińska K., Płaczkowska S., Prescha A., Pawełczyk K., Porębska I., Kosacka M., Pawlik-Sobecka L., Grajeta H. (2018). J. Trace Elem. Med. Biol..

[cit206] Zhu S., Liu Z., Qu R., Wang L., Li Q., Guan S. (2013). J. Magnesium Alloys.

[cit207] Stanford N., Atwell D. (2013). Metall. Mater. Trans. A.

[cit208] Azeem M., Tewari A., Ramamurty U. (2010). Mater. Sci. Eng., A.

[cit209] Xie B., Zhao M.-C., Tao J.-X., Zhao Y.-C., Yin D., Gao C., Shuai C., Atrens A. (2021). J. Mater. Res. Technol..

[cit210] Krawiec H., Stanek S., Vignal V., Lelito J., Suchy J. (2011). Corros. Sci..

[cit211] Kirkland N. T., Staiger M. P., Nisbet D., Davies C. H., Birbilis N. (2011). JOM.

[cit212] Yan Y., Cao H., Kang Y., Yu K., Xiao T., Luo J., Deng Y., Fang H., Xiong H., Dai Y. (2017). J. Alloys Compd..

[cit213] Cai S., Lei T., Li N., Feng F. (2012). Mater. Sci. Eng., C.

[cit214] Zhang S., Zhang X., Zhao C., Li J., Song Y., Xie C., Tao H., Zhang Y., He Y., Jiang Y. (2010). Acta Biomater..

[cit215] Calin M., Gebert A., Ghinea A. C., Gostin P. F., Abdi S., Mickel C., Eckert J. (2013). Mater. Sci. Eng., C.

[cit216] Kim H., Kim W. (2013). Corros. Sci..

[cit217] Fatmi M., Djemli A., Ouali A., Chihi T., Ghebouli M., Belhouchet H. (2018). Results Phys..

[cit218] Chye L.T., Zamzuri M. Z. M., Norbahiyah S., Ismail K. A., Derman M. N. B., Illias S. (2013). Adv. Mater. Res..

[cit219] Yuen C. K., Ip W. Y. (2010). Acta Biomater..

[cit220] Hidayah N. N., Abidin S. Z. (2018). Miner. Eng..

[cit221] Xie J., Zhang J., You Z., Liu S., Guan K., Wu R., Wang J., Feng J. (2021). J. Magnesium Alloys.

[cit222] Zhang J., Tong L., Xu C., Jiang Z., Cheng L., Kamado S., Zhang H. (2017). Mater. Sci. Eng., A.

[cit223] Liang M. J., Wu C., Ma Y., Wang J., Dong M., Dong B., Liao H. H., Fan J., Guo Z. (2021). Mater. Sci. Eng., C.

[cit224] Zhu T., Cui C., Zhang T., Wu R., Betsofen S., Leng Z., Zhang J., Zhang M. (2014). Mater. Des..

[cit225] Jung I.-H., Sanjari M., Kim J., Yue S. (2015). Scr. Mater..

[cit226] Meng J. , Sun W., Tian Z., Qiu X. and Zhang D., in Corrosion Prevention of Magnesium Alloys, Elsevier, 2013, pp. 38–60

[cit227] Ibrahim H., Esfahani S. N., Poorganji B., Dean D., Elahinia M. (2017). Mater. Sci. Eng., C.

[cit228] Krämer M., Schilling M., Eifler R., Hering B., Reifenrath J., Besdo S., Windhagen H., Willbold E., Weizbauer A. (2016). Mater. Sci. Eng., C.

[cit229] Ye C., Zheng Y., Wang S., Xi T., Li Y. (2012). Appl. Surf. Sci..

[cit230] Li T., He Y., Zhou J., Tang S., Yang Y., Wang X. (2018). Mater. Lett..

[cit231] Wang J. L., Xu J. K., Hopkins C., Chow D. H. K., Qin L. (2020). Adv. Sci..

[cit232] Jomaa M., Dieme D., Desrosiers M., Côté J., Fetoui H., Pelletier G., Nong A., Bouchard M. (2021). Toxicol. Lett..

[cit233] Pałasz A., Czekaj P. (2000). Acta Biochim. Pol..

[cit234] Cui G., Bi Z., Zhang R., Liu J., Yu X., Li Z. (2019). Chem. Eng. J..

[cit235] Pourhashem S., Vaezi M. R., Rashidi A., Bagherzadeh M. R. (2017). Corros. Sci..

[cit236] Kulyk B., Freitas M. A., Santos N. F., Mohseni F., Carvalho A. F., Yasakau K., Fernandes A. J., Bernardes A., Figueiredo B., Silva R. (2022). Crit. Rev. Solid State Mater. Sci..

[cit237] Cao X., Huang F., Huang C., Liu J., Cheng Y. F. (2019). Corros. Sci..

[cit238] Sochu W., Noraphaiphipaksa N., Manonukul A., Kanchanomai C. (2018). Int. J. Damage Mech..

[cit239] Schriver M., Regan W., Gannett W. J., Zaniewski A. M., Crommie M. F., Zettl A. (2013). ACS Nano.

[cit240] Hsieh Y.-P., Hofmann M., Chang K.-W., Jhu J. G., Li Y.-Y., Chen K. Y., Yang C. C., Chang W.-S., Chen L.-C. (2014). ACS Nano.

[cit241] Othman N. H., Ismail M. C., Mustapha M., Sallih N., Kee K. E., Jaal R. A. (2019). Prog. Org. Coat..

[cit242] Zhao G., Zhu H. (2020). Adv. Mater..

[cit243] Palaniappan N., Cole I., Kuznetsov A. (2020). RSC Adv..

[cit244] Chen Y., Ren B., Gao S., Cao R. (2020). J. Colloid Interface Sci..

[cit245] Zhang X., Zhou Y., Zhang J. (2017). Prog. Nat. Sci.: Mater. Int..

[cit246] Shin K., Acri T., Geary S., Salem A. K. (2017). Tissue Eng., Part A.

[cit247] Kundu C. K., Wang X., Song L., Hu Y. (2018). Prog. Org. Coat..

[cit248] Chen L., Tseng C.-M., Qiu Y., Yang J., Chang C.-L., Wang X., Li W. (2020). Surf. Coat. Technol..

[cit249] Cheng L., Liu C., Wu H., Zhao H., Wang L. (2022). J. Colloid Interface Sci..

[cit250] Ying Y., He P., Ding G., Peng X. (2016). Nanotechnology.

[cit251] Quezada-Renteria J. A., Chazaro-Ruiz L. F., Rangel-Mendez J. R. (2020). Carbon.

[cit252] Lynge M. E., van der Westen R., Postma A., Städler B. (2011). Nanoscale.

[cit253] Lee H., Dellatore S. M., Miller W. M., Messersmith P. B. (2007). Science.

[cit254] Yu R., Zhang H., Guo B. (2022). Nano-Micro Lett..

[cit255] Wang X.-p., Hou J., Chen F.-s., Meng X.-m. (2020). Sep. Purif. Technol..

[cit256] Pan C.-J., Hou Y., Wang Y.-N., Gao F., Liu T., Hou Y.-H., Zhu Y.-F., Ye W., Wang L.-R. (2016). Mater. Sci. Eng., C.

[cit257] Fang Z., Tu Q., Shen X., Yang X., Liang K., Pan M., Chen Z. (2022). Surf. Interfaces.

[cit258] Wang J.-l., Li B.-c., Li Z.-j., Ren K.-f., Jin L.-j., Zhang S.-m., Chang H., Sun Y.-x., Ji J. (2014). Biomaterials.

[cit259] Singer F., Schlesak M., Mebert C., Höhn S., Virtanen S. (2015). ACS Appl. Mater. Interfaces.

[cit260] Ma L., Qin H., Cheng C., Xia Y., He C., Nie C., Wang L., Zhao C. (2014). J. Mater. Chem. B.

[cit261] Ishizaki T., Shimada Y., Tsunakawa M., Lee H., Yokomizo T., Hisada S., Nakamura K. (2017). ACS Omega.

[cit262] Ishizaki T., Saito N. (2010). Langmuir.

[cit263] Xun X., Wan Y., Zhang Q., Gan D., Hu J., Luo H. (2020). Appl. Surf. Sci..

[cit264] Zang D., Zhu R., Wu C., Yu X., Zhang Y. (2013). Scr. Mater..

[cit265] Xu S., Wang Q., Wang N. (2021). Adv. Eng. Mater..

[cit266] Liu X., Zhang T. C., He H., Ouyang L., Yuan S. (2020). J. Alloys Compd..

[cit267] Liu Q., Chen D., Kang Z. (2015). ACS Appl. Mater. Interfaces.

[cit268] Zhang C., Zhang F., Song L., Zeng R., Li S., Han E. (2017). J. Alloys Compd..

[cit269] Yeganeh M., Mohammadi N. (2018). J. Magnesium Alloys.

[cit270] Li D.-W., Wang H.-Y., Liu Y., Wei D.-S., Zhao Z.-X. (2019). Chem. Eng. J..

[cit271] Zhao X., Wei J., Li B., Li S., Tian N., Jing L., Zhang J. (2020). J. Colloid Interface Sci..

[cit272] Wong T.-S., Kang S. H., Tang S. K., Smythe E. J., Hatton B. D., Grinthal A., Aizenberg J. (2011). Nature.

[cit273] Jiang D., Xia X., Hou J., Cai G., Zhang X., Dong Z. (2019). Chem. Eng. J..

[cit274] Zhang J., Gu C., Tu J. (2017). ACS Appl. Mater. Interfaces.

[cit275] Zang D., Zhu R., Zhang W., Yu X., Lin L., Guo X., Liu M., Jiang L. (2017). Adv. Funct. Mater..

